# Improved Computational Identification of Drug Response Using Optical Measurements of Human Stem Cell Derived Cardiomyocytes in Microphysiological Systems

**DOI:** 10.3389/fphar.2019.01648

**Published:** 2020-02-12

**Authors:** Karoline Horgmo Jæger, Verena Charwat, Bérénice Charrez, Henrik Finsberg, Mary M. Maleckar, Samuel Wall, Kevin E. Healy, Aslak Tveito

**Affiliations:** ^1^Department of Scientific Computing, Simula Research Laboratory, Oslo, Norway; ^2^Department of Bioengineering, College of Engineering, University of California, Berkeley, CA, United States; ^3^Department of Materials Science and Engineering, College of Engineering, University of California, Berkeley, CA, United States

**Keywords:** cardiac action potential model, computational inversion, cardiac ion channel blockade, human induced pluripotent stem cell derived cardiomyocytes, computational maturation, computational identification of drug response, voltage sensitive dye

## Abstract

Cardiomyocytes derived from human induced pluripotent stem cells (hiPSC-CMs) hold great potential for drug screening applications. However, their usefulness is limited by the relative immaturity of the cells’ electrophysiological properties as compared to native cardiomyocytes in the adult human heart. In this work, we extend and improve on methodology to address this limitation, building on previously introduced computational procedures which predict drug effects for adult cells based on changes in optical measurements of action potentials and Ca^2+^ transients made in stem cell derived cardiac microtissues. This methodology quantifies ion channel changes through the inversion of data into a mathematical model, and maps this response to an adult phenotype through the assumption of functional invariance of fundamental intracellular and membrane channels during maturation. Here, we utilize an updated action potential model to represent both hiPSC-CMs and adult cardiomyocytes, apply an IC50-based model of dose-dependent drug effects, and introduce a continuation-based optimization algorithm for analysis of dose escalation measurements using five drugs with known effects. The improved methodology can identify drug induced changes more efficiently, and quantitate important metrics such as IC50 in line with published values. Consequently, the updated methodology is a step towards employing computational procedures to elucidate drug effects in adult cardiomyocytes for new drugs using stem cell-derived experimental tissues.

## Introduction

The development of human induced pluripotent stem cells (hiPSCs) opens promising avenues of investigation into a wide variety of fundamental questions in cell physiology and beyond [for recent reviews, see, e.g., (Yoshida and Yamanaka, 2017; Di Baldassarre et al., 2018; Ye et al., 2018)]. One of the more immediately tractable applications of hiPSCs is the creation of specific human cell and tissue samples to augment drug discovery and development pipelines. These pipelines have traditionally relied on animal models in key areas of testing, but are limited by significant physiological differences between animal and human cells [see, e.g., (Mathur et al., 2016; Fine and Vunjak-Novakovic, 2017; Yoshida and Yamanaka, 2017; Ye et al., 2018)]. These differences, both at the genetic and proteomic levels, give rise to distinctly non-human system dynamics, for example, a mouse’s heart rate is much more rapid than a human’s (∼600 bpm vs. ∼60 bpm), such that it is often difficult to translate drug effects from one species to another [see, e.g., (Mathur et al., 2016; Fine and Vunjak-Novakovic, 2017; Yoshida and Yamanaka, 2017; Ye et al., 2018)].

By using hiPSC-derived cells, it is possible to measure drug effects directly in human-based systems, and therapeutics can eventually, in principle, be tested and adjusted at the level of the individual patient. This hiPSC-based, patient-centric approach opens up great possibilities for drug development, both in terms of the scope of illnesses approachable, including disorders caused by rare mutations, as well as improved safety by the early identification of drug side effects in *human* cells. Nevertheless, hiPSCs are also associated with a variety of scientific challenges that must be resolved to realize the full potential of the technology [see, e.g., (Mathur et al., 2015; Mathur et al., 2016; Mora et al., 2017; Christensen et al., 2018; Ronaldson-Bouchard et al., 2018; Zhao et al., 2018)].

Maturity of generated cells and tissues is one of these key challenges, a prominent example being the maturation of hiPSC-derived cardiomyocytes (hiPSC-CMs) (Chen et al., 2016). Human cardiomyocytes develop over many years [see (Hille, 2001), ch. 21], and during this period the density of specific ion channels changes significantly, due both to increased area of the cell membrane and proliferation of membrane channels [see, e.g., (Sontheimer et al., 1992; Moody and Bosma, 2005; Bedada et al., 2016)]. Therefore, the physiological response of immature hiPSC-CMs to a drug cannot necessarily be used to infer the properties of the drug, nor the response of adult human cardiomyocytes. Even if it is known exactly how a drug affects an hiPSC-CM, it is difficult to deduce its effect on adult cells; direct interpretation may in fact lead to both false positives and false negatives [see (Liang et al., 2013; Mathur et al., 2015)].

In (Tveito et al., 2018), we used mathematical modeling of cardiac cell dynamics to address these challenges associated with the application of hiPSC-CMs. Such mathematical modeling of the cardiac action potential (AP) remains an active area of research, and sophisticated models have been developed in order to accurately simulate both single cells and cardiac tissue dynamics [see e.g., (CellML Model Repository; Rudy and Silva, 2006; Grandi et al., 2010; O’Hara et al., 2011; Rudy, 2012; Franzone et al., 2014; Qu et al., 2014; Edwards and Louch, 2017; Quarteroni et al., 2017; Tveito et al., 2017)]. We presented an algorithm for inverting experimental measurements of the membrane potential and the cytosolic calcium (Ca^2+^) concentration in order to obtain parameters for a mathematical model of the hiPSC-CM AP. We then demonstrated how this model of hiPSC-CMs can be mapped to an AP model representing adult cells. We were able to estimate the effect of a drug on essential ion currents for hiPSC-CMs as based on measurements from a microphysiological system (Mathur et al., 2015), and then to map this effect onto the adult cardiomyocyte AP model. The combination of these two methods permitted, in principle, to deduce drug effects on adult human cardiomyocytes as based on measurements of hiPSC-CMs in a microphysiological system.

**Figure 1:**
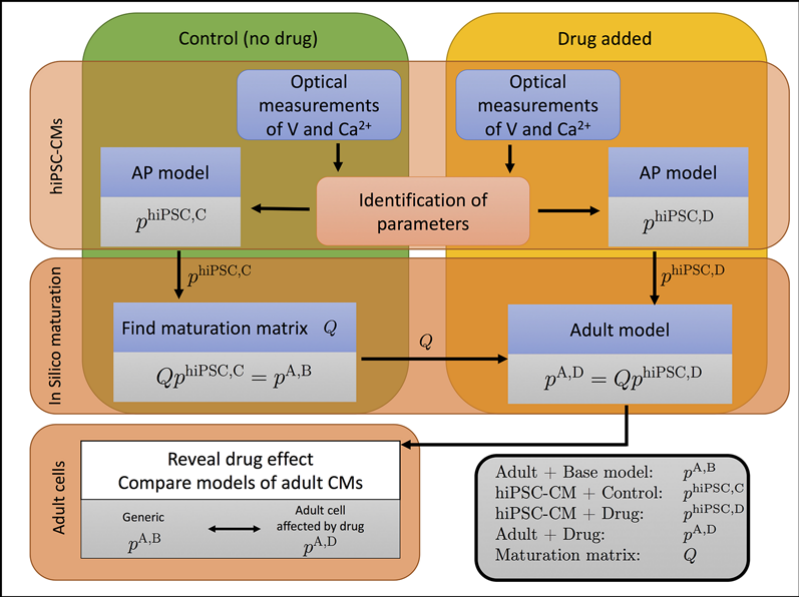
The effect of a drug on adult cardiomyocytes can be identified by the process illustrated. The cytosolic calcium concentration (Ca^2+^) and the membrane potential (V) are measured in a microphysiological system (Mathur et al., 2015; Mathur et al., 2016) of hiPSC-CMs. Data are collected when no drug has been applied (control, C) and when a drug has been applied (D). The data are used to parameterize a model for both cases, represented by the parameter vectors *p*^hiPSC,C^ and *p*^hiPSC,D^ for the control and drugged cases, respectively. The control parameter vector *p*^hiPSC,C^ is used to define the maturation matrix *Q* such that *Qp*^hiPSC,C^ = *p^A,B^*, where *p^A,B^* is the parameter vector of a generic base model of adult human cardiomyocytes. By comparing the adult parameter vectors for the control and drugged cases, the effect of the drug can be identified.

The overall method developed in (Tveito et al., 2018) is illustrated in **Figure 1**. In this procedure, we take optical measurements using fluorescent dyes in a microphysiological system to define relative traces of the membrane potential and cytosolic Ca^2+^ concentration for cells under normal media conditions and in the presence of drugs. We then define a mathematical model for the control (undrugged) cases by identifying parameters denoted by *p*^hiPSC,C^ (hiPSC is for hiPSC-derived, C is for control) in an AP model that matches the experimental waveforms. Using this model of hiPSC-CMs, we then define a maturation matrix *Q* such that *Qp*^hiPSC,C^ = *p*^A,B^, where *p*^A,B^ (A is for adult, B is for base) are *known* parameters representing a generic AP model of an adult human cardiomyocyte. Here, the matrix *Q* represents the developmental change in ion channel density and geometry from hiPSC-CMs to adult cardiomyocytes, independent of drug effects on individual channels.

Next, experimental traces of the membrane potential and cytosolic Ca^2+^ concentration are taken for the same cells in the presence of drugs, and these traces are used to define a new parameter-vector *p*^hiPSC,D^ (hiPSC-CM, Drug) that matches the data. This new parameterization gives us information about what modeled channels have been altered by the drug. Then, by assuming that the drug affects *every individual ion channel* in the same manner for the hiPSC-CMs and the adult cells, the parameter vector for the adult case is given by *p*^A,D^ = *Qp*^hiPSC,D^. *Hence, we can find an AP model for adult human cardiomyocytes under the influence of the drug, even though only the effect for hiPSC-CMs has been measured*.

The present report aims to present a number of modifications to improve the accuracy and reliability of these methods. First, using the AP models of Grandi et al. (2010), O’Hara et al. (2011), Paci et al. (2013), Paci et al. (2015), Paci et al. (2017), and Paci et al. (2018) as a basis, we have derived a new AP model to improve representation of experimental data. As our inversion algorithm is based on conducting a huge number of simulations with varying parameter values, it is essential to have a model that is stable with respect to perturbations of the parameters. Therefore, the new model is designed for improved stability. In particular, the model of the intracellular Ca^2+^ dynamics has been modified to avoid instabilities in the balance between the influx and efflux of Ca^2+^ to the sarcoplasmic reticulum (SR).

In addition, our aim has been to create models that can be mapped back and forth between hiPSC-CMs and adult cardiomyocytes. A vital modeling assumption has been that the individual channels are the same in these two cases, and that only channel density should change. However, existing AP models are not derived with such a mapping in mind, and models of identical single channel dynamics vary significantly among models. Therefore, we have derived a new AP model which strictly adheres to the principle that every current (and flux) should be written as a product of the ion channel density and the dynamics of a single channel; identical ion channels are represented by identical mathematical models. Consequently, the mathematical representation of *a single channel* is the same for the hiPSC-CMs and adult cardiomyocytes in the novel AP model presented here.

Finally, we present a new method for inverting experimental data into parameters for the AP model by introducing a continuation-based approach, searching for optimal parameters by gradually moving from known parameters to the parameters we want to identify. Continuation methods are well developed in scientific computing [see e.g., (Keller, 1987; Allgower and Georg, 2012)] and offer significant computational savings to find optimal solutions.

In this manuscript, we first motivate and describe the approaches outlined above. We then evaluate these methods with respect to accuracy using simulated data. Subsequently, the new methods are used to identify the effect of five well-characterized drugs based on optical measurements of hiPSC-CMs. In all cases considered, the predicted effects are consistent with known drug effects, lending credence to the principle that novel drug effects on adult cardiomyocytes could reliably be estimated using measurements of hiPSC-CMs and the described methodology.

## Methods

Here, we offer a detailed presentation of all steps illustrated in **Figure 1**. First, we present the derivation of a new AP model. Next, we describe the inversion method used in our computations. Finally, we discuss how to characterize the identifiability of the parameters involved in the inversion as based on singular value decomposition (SVD) of model currents.

### The Base Model

As noted above, we aim to define an AP model that can be scaled from very early stages of human development (days) to fully developed adult cardiomyocytes. To review, for one specific membrane current, we assume that the only difference between the hiPSC-CM and adult cases is that the number of channels and the membrane area has changed; thus, the density of the specific ion channel carrying the current has changed, but the properties of every individual channel remains the same. The same principle holds for the intracellular Ca^2+^ machinery; the individual channels and buffers remain the same, but both the intracellular volumes and the number of channels change from hiPSC-CMs to adult cardiomyocytes. Our model will, therefore, be based on models of single ion channel dynamics and *only the density of these single channels will change*. When a drug is involved, we assume that the effect of the drug on a *single channel* is the same in the hiPSC-CM and adult cases, and therefore one can use the effect in the hiPSC-CM case to estimate the effect for the adult case.

### Modeling the Membrane Currents

The standard model [see, e.g., (Izhikevich, 2007; Plonsey and Barr, 2007; Ermentrout and Terman, 2010; Sterratt et al., 2011)] of the membrane potential of an excitable cell is given by the equation

(1) $$\frac{dv}{dt}=-\sum_{x}I_{x},$$

where *v* is the membrane potential (in mV), and *I_x_* are the membrane currents through ion channels of different types, as well as pumps and exchangers located on the cell membrane.

These currents are all given in units of A/F, and may be written on the form

(2) $$I_{x}=\frac{N_{x}}{AC_{m}}i_{x,}$$

where *N_x_* is the number of channels of type *x* on the cell membrane, *A* is the area of the cell membrane (in μm^2^) and *C_m_* is the specific capacitance of the cell membrane (in pF/μm^2^). Furthermore, *i_x_* represents the average current through a single channel of type *x* (in pA). For voltage-gated ion channels, this average single-channel current is given on the form

(3) $$i_{x}=g_{0}^{x}o_{x}(v-E_{x}),$$

where $g_{0}^{x}$ is the conductance of a single open channel (in nS), *E_x_* is the equilibrium potential of the channel (in mV), and *o_x_* is the unitless open probability of the channel. Note that in models given on this form, it is common to consider a lumped parameter *g_x_*, given by

$$g_{x}=\frac{N_{x}}{AC_{m}}g_{0}^{x},$$

and parameters of this type are given for each of the ion channels considered in the base model in the **Supplementary Information**. For membrane pumps and exchangers, the single-channel current is given on a similar form. The specific currents included in the model will be described below.

#### Scaling of the Membrane Currents

As mentioned above, we assume that the specific membrane capacitance and the ion channels responsible for each of the membrane currents are the same during different stages of development for the cell, but that the number of ion channels, *N_x_*, and the membrane area, *A*, may differ. Therefore, currents can be mapped from one stage of development, *S*_1_, to another stage of development, *S*_2_, simply by adjusting the channel density of the currents.

More specifically, for the formulation (1)–(2), this means that we assume that the parameter *C_m_* and the expressions for the single-channel currents, *i_x_*, are the same for *S*_1_ and *S*_2_, but that the channel density $\frac{N_{x}}{A}$ can be different. Let $A_{x}^{S_{1}},A_{x}^{S_{2}}$ and $N_{x}^{S_{1}},N_{x}^{S_{2}}$ denote the membrane area and number of channels of type *x* for the *S*_1_ and *S*_2_ cases, respectively. Furthermore, let λ*_x_* represent the change of channel density in the sense that

(4) $$\frac{N_{x}^{S_{1}}}{A_{x}^{S_{1}}}=(1+\lambda_{x})\frac{N_{x}^{S_{2}}}{A_{x}^{S_{2}}}.$$

Now, the S_1_ and S_2_ currents are related according to

(5) $$I_{x}^{S_{1}}=\frac{N_{x}^{S_{1}}}{A_{x}^{S_{1}}C_{m}}i_{x}=(1+\lambda_{x})\frac{N_{x}^{S_{2}}}{A_{x}^{S_{2}}C_{m}}i_{x}=(1+\lambda_{x})I_{x}^{S_{2}},$$

for each of the currents *x*.

#### The Base Model is the Generic Adult Model

Based on these considerations, it is convenient to define *one* default base model from which all other models are derived to simplify a mapping procedure between different development stages.

Defining a base model as representing hiPSC-CMs, from which adult cardiomyocytes subsequently develop, may seem to be a natural choice. However, in the scheme illustrated in **Figure 1**, there is only one fixed model—the generic adult model—while all other models will change depending on the experimental measurements. For simplicity, we, therefore, define the generic adult model to be the *default base model*, and scale all other models relative to this model.

#### Main Currents Present in Human Cardiomyocytes

Modern models of human cardiomyocytes are complex and the models for the individual currents are based on years of experience using patch-clamp measurements. In the formulation (1), our aim has been to include the main currents present in human cardiomyocytes, but to keep the number of currents as low as feasible in order to keep the base model relatively simple. The experimental inputs in the present methodology are optically-derived, and data based on sensitive dyes are not expected to be able to uncover equally fine details of the dynamics as compared to traditional electrophysiological measurements derived *via* patch clamp. It is, therefore, reasonable to represent the data using simpler models. Our choice of currents is based on the O’Hara et al. (2011) model and the Grandi et al. (2010) model for human adult ventricular cardiomyocytes, in addition to the Paci et al. (2013) model for hiPSC-CMs. Furthermore, we have focused on including currents considered to be critical for depolarization and repolarization of the AP and, therefore, those typically investigated for response to drugs [see, e.g., (Crumb et al., 2016)].

In (Crumb et al., 2016), the fast sodium current, *I*_Na_, the late sodium current, *I*_NaL_, the L-type Ca^2+^ current, *I*_CaL_, the transient outward potassium current, *I*_to_, the rapid and slow delayed rectifier potassium currents, *I*_Kr_ and *I*_Ks_, and the inward rectifier potassium current, *I*_K1_, were investigated for their drug responses, and we have included each of these currents in our model. In addition, we have included the sodium-potassium pump, *I*_NaK_, the sodium-calcium exchanger, *I*_NaCa_, the Ca^2+^ pump, *I*_pCa_, the background Ca^2+^ current, *I*_bCa_, and the background chloride current, *I*_bCl_, as they all appear to have a significant effect on the computed AP and Ca^2+^ transient of the Grandi et al. (2010) model. Furthermore, we have included the hyperpolarization-activated cyclic nucleotide-gated funny current, *I*_f_. While this current is very small for adult ventricular cardiomyocytes, it is substantial for hiPSC-CMs (Garg et al., 2018). The formulation used for each of the currents is given in the **Supplementary Information**. The formulations are based on those of the currents in the Paci et al. (2013), the Grandi et al. (2010), and the O’Hara et al. (2011) models, and we have chosen formulations that seems to work well for both the hiPSC-CM and adult cases and that are able to provide good fits for our considered data of hiPSC-CMs.

### Modeling Intracellular Ca^2+^ Dynamics

In addition to the membrane potential, we also want the base model to represent the dynamics of the intracellular Ca^2+^ concentration. We consider the following five intracellular compartments [based on (Grandi et al., 2010)]:

1. The dyad, representing the small cytosolic subspace between the L-type Ca^2+^ channels and the ryanodine receptors (RyRs),
2. The subsarcolemmal space, representing the remaining part of the cytosolic space that is located close to the membrane,
3. The bulk cytosolic space,
4. The junctional sarcoplasmic reticulum (jSR), representing the part of the SR that is close to the RyR-channels,
5. The network sarcoplasmic reticulum (nSR), representing the remaining part of the SR.

**Figure 2:**
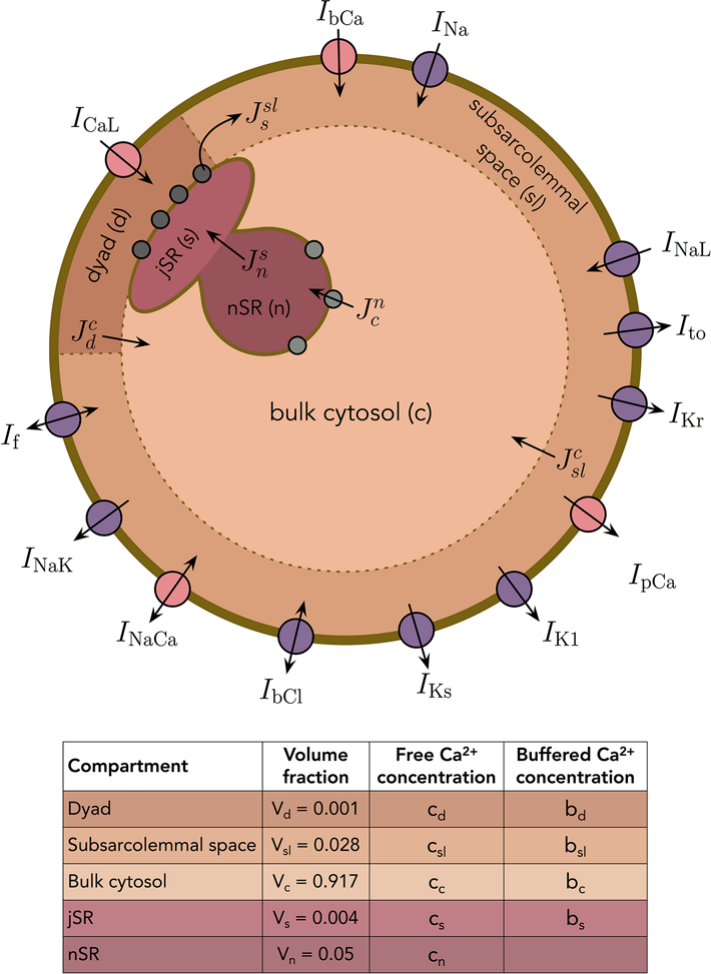
Membrane currents, Ca^2+^ fluxes and intracellular compartments of the base model. The volume fractions of the compartments are based on (Grandi et al., 2010).

The Ca^2+^ concentrations and volume fractions defined for each of these compartments are given in **Figure 2**. In all compartments except the nSR, we consider both the concentration of free Ca^2+^ and the concentration of Ca^2+^ bound to a buffer. The Ca^2+^ concentration in the extracellular space is assumed to remain constant. The intracellular Ca^2+^ fluxes between compartments are illustrated in **Figure 2**, and the model takes the form

$$\begin{array}{l} \begin{array}{cc} \frac{dc_{d}}{dt}=\frac{1}{V_{d}}(J_{CaL}-J_{d}^{b}-J_{d}^{c}), & \frac{db_{d}}{dt}=\frac{1}{V_{d}}J_{d}^{b},\\ \;\frac{dc_{sl}}{dt}=\frac{1}{V_{sl}}(J_{e}^{sl}-J_{sl}^{c}-J_{sl}^{b}+J_{s}^{sl}), & \frac{db_{sl}}{dt}=\frac{1}{V_{sl}}J_{sl}^{b},\\ \;\frac{dc_{c}}{dt}=\frac{1}{V_{c}}(J_{sl}^{c}+J_{d}^{c}-J_{c}^{n}-J_{c}^{b}), & \frac{db_{c}}{dt}=\frac{1}{V_{c}}J_{c}^{b},\\ \frac{dc_{s}}{dt}=\frac{1}{V_{s}}(J_{n}^{s}-J_{s}^{sl}-J_{s}^{b}), & \frac{db_{s}}{dt}=\frac{1}{V_{s}}J_{s}^{b}, \end{array}\\ \;\frac{dc_{n}}{dt}=\frac{1}{V_{n}}(J_{c}^{n}-J_{n}^{s}). \end{array}\;$$

See Section S2.1 in the **Supplementary Information** for a derivation of these equations.

#### Definition of Ca^2+^ Fluxes

Every model flux *J_x_* representing the flux through a type of channel can be written on the form

$$J_{x}=\frac{N_{x}}{V_{\text{cell}}}j_{x},$$

where *N_x_* is the number of channels of type *x*, *V*_cell_ is the cell volume (in L) and *j_x_* is the average flux through a single channel of type *x* (in mmol/ms). Below, we will describe the formulation chosen for the flux through the RyR channels. Definitions of each of the remaining fluxes are specified in the **Supplementary Information**.

#### Modeling Release From the SR

In our model of Ca^2+^ dynamics, we deviate from previous modeling approaches in two specific ways:

1. Ca^2+^ is released through RyR channels from the SR directly to the subsarcolemmal space (SL) and not to the dyad.
2. Release of Ca^2+^ through the RyR channels is a product of two factors; one factor models the open probability of the RyR channels, whereas the other models the availability of channels that can be opened. We assume that each channel can only process a certain amount of Ca^2+^ before it deactivates.

We will see below that these two modeling assumptions lead to a model that exhibits two key physiological features of Ca^2+^ release from the SR of cardiomyocytes, so-called *high gain* and *graded release* (see Section S2.2 in the **Supplementary Information** for explanations of these terms).

##### Flux through RyRs ($J_{s}^{sl}$)

As we will employ the base model for several different parameter combinations, the model for the RyR flux must be stable, in the sense that careful tuning of the model is not requisite to ensure reasonable activation and deactivation of the RyRs.

As outlined above, we let the Ca^2+^ released from the SR enter the SL space rather than the dyad. This is done in order to achieve graded release (see the **Supplementary Information**), in the sense that the amount of Ca^2+^ released from the SR through the RyRs should depend directly upon the amount of Ca^2+^ entering the cell through L-type Ca^2+^ channels. If the model were to be formulated such that Ca^2+^ released from the jSR instead entered the dyad, it would be difficult to distinguish the increase in dyadic Ca^2+^ concentration resulting from L-type Ca^2+^ channel flux as opposed to release via RyRs. Directing the RyR flux into the SL, the concentration change in the dyad is almost exclusively due to the influx through L-type Ca^2+^ channels, and by letting the flux through the RyRs depend on the Ca^2+^ concentration in the dyad, we achieve graded release.

Furthermore, a common modeling approach for the RyRs is to govern inactivation by a decreased jSR concentration [see e.g., (Sobie et al., 2010)]. However, for large variations in parameter values, this may lead to model instabilities, because the jSR concentration depends upon the balance between the flux through the SERCA pumps and the RyRs, which depend upon the balance between the Ca^2+^ fluxes in and out of the cell. In order to avoid an RyR model whose inactivation mechanism depends on the jSR concentration, we instead introduce a new assumption that some RyRs are only able to carry a given amount of Ca^2+^ ions during each AP.

We then assume that a small portion of the RyR channels are always open (type 0), while the remaining channels (type 1) are activated by an increased dyadic Ca^2+^ concentration and are inactivated after they have transported a given amount of Ca^2+^ ions. Therefore, the total flux through the RyRs may be expressed as

(6) $$J_{s}^{sl}=J_{\text{RyR}}+J_{\text{leak}},$$

where *J*_RyR_ represents the flux through the RyR channels of type 1 and *J*_leak_ represents the flux through the RyR channels of type 0. We assume that the flux through the two types of RyR channels are given by expressions of the form

(7) $$J_{\text{RyR}}=\frac{Mp}{V_{\text{cell}}}j_{\text{RyR}},$$

(8) $$J_{\text{leak}}=\frac{M_{0}}{V_{\text{cell}}}j_{\text{RyR}},$$

where *j*_RyR_ denotes the flux through a single open RyR channel (in mmol/ms) and *V*_cell_ denotes the total cell volume (in L). In addition, *M*_0_ denotes the number of RyR channels that are always open (type 0), *M* denotes the number of available RyR channels of type 1, and *p* is the open probability of the channels of type 1. The single channel flux through the RyRs is given by

(9) $$j_{\text{RyR}}=\alpha_{\text{RyR},0}(c_{s}-c_{sl}),$$

where *α*_RyR,0_ (in L/ms) represents the rate of release. Furthermore, the open probability of the RyR channels of type 1 is modeled by a simple function that increases sigmoidally with the dyadic Ca^2+^ concentration, *c_d_*, based on the model in (Friel, 1995):

(10) $$p=\frac{c_{d}^{3}}{c_{d}^{3}+\kappa_{\text{RyR}}^{3}}.$$

We let the total number of RyR channels of type 1 be given by *N*_RyR_ and the total number of RyR channels of type 0 be given by

(11) $$M_{0}=\gamma_{\text{RyR}}N_{\text{RyR}}.$$

In other words, the total number of RyR channels (of both types) is given by (1+γ_RyR_)*N*_RyR._

We assume that every RyR of type 1 is able to transport a fixed amount of *Z* Ca^2+^ ions during an AP. After *Z* ions have been transported, the channel becomes inactivated. However, we assume that as the dyadic Ca^2+^ concentration, *c_d_*, returns to rest and the open probability, *p*, consequently decreases, the inactivated channels gradually recover from inactivation. We let the number of available channels of type 1 be governed by

(12) $$\frac{dM}{dt}=-\frac{V_{\text{cell}}}{Z}J_{\text{RyR}}+\frac{\eta_{\text{RyR}}}{p}(N_{\text{RyR}}-M).$$

Here, the first term dominates for large values of *p*, driving *M* towards zero as more Ca^2+^ is transported through the RyR channels of type 1. Furthermore, for small values of *p* (i.e., at rest), the second term dominates and drives *M* towards the maximum value *N*_RyR_.

In order to reduce the number of free parameters in the model, we define a scaled variable *r*, defined as $r=\frac{M}{N_{\text{RyR}}}$, and divide both sides of equation (12) by *N*_RyR_. The equation then reads

(13) $$\frac{dr}{dt}=-\frac{J_{\text{RyR}}}{\beta_{\text{RyR}}}+\frac{\eta_{\text{RyR}}}{p}(1-r),$$

where

(14) $$\beta_{\text{RyR}}=\frac{N_{\text{RyR}}}{V_{\text{cell}}}Z.$$

Inserting *M = rN*_RyR_ into (7) and defining

(15) $$\alpha_{\text{RyR}}=\frac{N_{\text{RyR}}}{V_{\text{cell}}}\alpha_{\text{RyR,}0},$$

we get the following expression for active RyR flux

(16) $$J_{\text{RyR}}=p\cdot r\cdot \alpha_{\text{RyR}}(c_{s}-c_{sl}),$$

where we recall that

(17) $$p=\frac{c_{d}^{3}}{c_{d}^{3}+\kappa_{\text{RyR}}^{3}}.$$

Moreover, inserting (11) and (15) into (8), we obtain

(18) $$J_{leak}=\gamma_{\text{RyR}}\cdot \alpha_{\text{RyR}}(c_{s}-c_{sl}).$$

##### Scaling the RyR flux

 When considering cells of different levels of maturity, we assume that the number of RyRs and the cell volume may be different, but that the function of a single RyR channel is the same for different levels of maturity. We also assume that the ratio between RyR channels of type 0 and 1, γ_RyR_, and the number of Ca^2+^ ions that each RyR channel of type 1 can transport, *Z*, is the same for the different maturity levels. Considering the model (13)–(18), this means that the only adjustment necessary between two maturity levels *S*_1_ and *S*_2_ is an adjustment of the density $\frac{N_{\text{RyR}}}{V_{\text{cell}}}$ in the definition of α_RyR_ and β_RyR_. We, therefore, introduce a scaling factor λ_RyR_ such that

(19) $$\frac{N_{\text{RyR}}^{S_{1}}}{V_{\text{cell}}^{S_{1}}}=(1+\lambda_{\text{RyR}})\frac{N_{\text{RyR}}^{S_{2}}}{V_{\text{cell}}^{S_{2}}},$$

and represent this adjustment of the RyR density in the model by scaling *α*_RyR_ and *β*_RyR_ by

(20) $$\alpha_{\text{RyR}}^{S_{1}}=(1+\lambda_{\text{RyR}})\alpha_{\text{RyR}}^{S_{2}},$$

(21) $$\beta_{\text{RyR}}^{S_{1}}=(1+\lambda_{\text{RyR}})\beta_{\text{RyR}}^{S_{2}},$$

where superscript *S*_1_ and *S*_2_ denote the S_1_ and S_2_ versions of the parameters, respectively.

### Inversion of Optical Measurements

The inversion procedure, used to construct base model representations of data obtained from optical measurements of the AP and Ca^2+^ transient of hiPSC-CMs, is described below. First, in Section *Optical Measurements*, we describe how optical measurements of hiPSC-CMs are obtained. Next, in Section *Definition of Adjustment Factors*, we describe how adjustment factors λ are set up to represent control (non-drugged) cells from different data sets. In Section *IC50 Modeling of Drug Effects*, we describe how the effect of a drug is modeled using IC50 values and corresponding factors, denoted by *ε*. The aim of the inversion procedure is to find optimal parameter vectors λ and ε so that the model parameterized by λ and *ε* aligns to the measured data as best possible. This is explained in more detail in Section *Coupled Inversion of Data From Several Doses*. In Section *Properties of the Cost Function*, we describe the cost function constructed to measure the difference between the model and the data. Finally, in Section *A Continuation-Based Minimization Method*, we describe the continuation-based minimization method used to minimize the cost function in our computations.

#### Optical Measurements

Using previously developed techniques (Mathur et al., 2015), cardiac microphysiological systems derived from a single line of hiPSCs were loaded and matured prior to drug exposure. The resulting tissues consisted of approximately 90% cardiomyoctyes, with a small population of stromal support cells. On the day upon which studies were performed, freshly measured drugs (Nifedipine, Lidocaine, Cisapride, Flecainide, and Verapamil) were dissolved into DMSO or media and serially diluted. Each concentration of the drug to be tested was preheated for 15–20 min in a water bath at 37°C and subsequently sequentially injected in the device. At each dose, after 20 min of exposure, the drug’s response on the microtissue was recorded using a Nikon Eclipse TE300 microscope fitted with a QImaging camera. Fluorescent images were acquired at 200 frames per second using filters to capture GCaMP and BeRST-1 fluorescence as previously described. Images were obtained across the entire chip for 6–8 sec at a resolution of 511 x 222 square 1.3 micron pixels. Excitation was paced at 1 Hz, to capture multiple beats for processing.

Fluorescence videos were analyzed using custom Python software utilizing the open source Bio-Formats tool to produce characteristic AP and Ca^2+^ waveforms for each chip and tested drug dose. Briefly, for each analysis, the fluorescent signal was averaged over the entire microtissue. The signal was then smoothed using a 3-point median filter, and five to seven individual action potentials or calcium transients were overlayed by aligning the maximum dF/dt and then averaged into a single transient. For each drug escalation study, we chose the single series with the most continuity between control cases and subsequent drug doses for both AP and Ca^2+^ transient for inversion and mapping analysis.

#### Definition of Adjustment Factors

In order to make base model representations of control cells from different data sets, we must define adjustment factors λ for a base model parameter set. These adjustment factors represent alterations of the channel densities and geometry of the cells under consideration, as explained above. For example, for each membrane channel type *x*, the adjustment factor λ*_x_* is defined as

(22) $$\frac{N_{x}}{A}=(1+\lambda_{x})\frac{N_{x}^{b}}{A_{b}},$$

where $\frac{N_{x}}{A}$ is the channel density on the cell membrane for the fitted model and $\frac{N_{x}^{b}}{A^{b}}$ is the channel density in the default base model. We generally consider adjustment factors for the membrane channel densities for all the currents of the model, i.e., λ_Na_, λ_NaL_, λ_CaL_, λ_to_, λ_Kr_, λ_Ks_, λ_K1_, λ_NaCa_, λ_NaK_, λ_pCa_, λ_bCl_, λ_bCa_, and λ_f_, although some of the factors are fixed in some cases (see Section *Identifiability of the Currents in the hiPSC-CM Base Model*).

For the density of an intracellular channel type *x*, the adjustment factor λ*_x_* is similarly defined as

(23) $$\frac{N_{x}}{V_{\text{cell}}}=(1+\lambda_{x})\frac{N_{x}^{b}}{V_{\text{cell}}^{b}},$$

where $\frac{N_{x}}{V_{\text{cell}}}$ and $\frac{N_{x}^{b}}{V_{\text{cell}}^{b}}$ are the channel densities for the fitted model and the default base model, respectively. We consider the adjustment factors λ_RyR_ and λ_SERCA_ for intracellular channel densities, and the factors $\lambda_{B}^{d}$, $\lambda_{B}^{sl}$, $\lambda_{B}^{c}$ and $\lambda_{B}^{s}$ for intracellular calcium buffers (see the **Supplementary Information**). In addition, we consider adjustments to the intracellular diffusion coefficients, $\lambda_{d}^{c}$, $\lambda_{sl}^{c}$, and $\lambda_{n}^{c}$ (see the **Supplementary Information**). In order to reduce the number of free parameters to be determined in the inversion procedure for different control data, we assume that the buffer concentrations change at the same rate in all intracellular compartments, so that we only consider a single adjustment factor

(24) $$\lambda_{B}^{d}=\lambda_{B}^{sl}=\lambda_{B}^{c}=\lambda_{B}^{s}:=\lambda_{B}.$$

Similarly, we assume that the intracellular diffusion coefficients change at the same rate, so that

(25) $$\lambda_{d}^{c}=\lambda_{sl}^{c}=\lambda_{n}^{s}:=\lambda_{\alpha }.$$

Furthermore, because we wish to avoid ending up with unrealistic values of the surface-to-volume ratio, χ, we assume that the scaling factor for the cell surface-to-volume ratio varies little between data sets and only employ two different values of χ in the computations. We use the value χ = 0.6 *μ*m^˗1^ for adult cells and the value χ = 0.9 *μ*m^˗1^ for hiPSC-CMs, based on the values used in the Grandi et al. AP model for adult cardiomyocytes (Grandi et al., 2010) and the Paci et al. AP model for hiPSC-CMs (Paci et al., 2013). Note here that t-tubules (i.e., invaginations of the cell membrane extending into the center of the cell) are present for adult ventricular cardiomyocytes (Orchard et al., 2009), and this is incorporated into the adult version of χ by increasing the cell surface area by a factor of about two compared to the geometrical surface of the cylinder shape of the cell [see e.g., (Luo and Rudy, 1994)]. For hiPSC-CMs, t-tubules are believed to be absent or underdeveloped [see e.g., (Di Baldassarre et al., 2018; Jiang et al., 2018)], and in our choice of χ for hiPSC-CMs, we have assumed that t-tubules are not present for hiPSC-CMs.

#### IC50 Modeling of Drug Effects

Following previous modeling of channel blockers [see, e.g., (Brennan et al., 2009; Davies et al., 2012; Zemzemi et al., 2013; Paci et al., 2015)], we model the dose-dependent effect of a drug by scaling the channel conductances according to

(26) $$g_{i}^{D}=\frac{1}{1+\frac{D}{IC50_{i}}}g_{i}^{C},$$

where $g_{i}^{D}$ is the conductance of channel *i* in the presence of a drug with concentration *D*, IC50_*i*_ is the drug concentration that leads to 50% block of channel *i*, and $g_{i}^{C}$ is the channel conductance in the control case (i.e., in the absence of drugs). Specifically, this means that if the drug concentration *D* equals the IC50 value, we have $g_{i}^{D}=\frac{1}{2}g_{i}^{C}$.

It should be mentioned that a drug may certainly affect a channel in a more complex manner than is assumed here. The effect of drugs can realistically be represented by introducing new states in Markov models of the ion channel. In such models, the transition rates between different model states are able to represent the properties of drugs [see e.g., (Clancy et al., 2007; Tveito et al., 2011; Tveito and Lines, 2016; Tveito et al., 2018)]. Although Markov model representations of drug effects are more versatile and realistic than the simple blocking assumption employed here (Tveito et al., 2017), it would greatly increase the complexity of the inversion process, as many more parameters would have to be computed.

From (26), we see that for a given drug dose *D* > 0, the effect of the drug would increase if the IC50 value were decreased, and the effect of the drug would be very small if the IC50 value were much larger than the considered dose. In the continuation-based minimization method applied in our computations (see the section *A Continuation-Based Minimization Method* below), it is most practical to deal with parameters that are small when no change occurs and large when large changes occur. Therefore, we introduce the parameters

(27) $$\varepsilon_{i}=\frac{1}{IC50_{i}}.$$

Here, a small value of *ε_i_* represents small effects of a drug while a large value of *ε_i_* represents large effects, and channel blocking is given by

(28) $$g_{i}^{D}=\frac{1}{1+D\varepsilon_{i}}g_{i}^{C}.$$

In our computations, we assume that the considered drugs block either *I*_CaL_, *I*_NaL_, *I*_Kr_, or a combination of these currents, and we therefore only consider the *ε*-parameters *ε*_CaL_, *ε*_NaL_, *ε*_Kr_.

#### Coupled Inversion of Data From Several Doses

The control data obtained from different optical experiments tend to vary significantly, and in order to be able to accurately estimate the drug effect from these measurements, the λ-parameters must be tuned so that the control model fits the control data as best possible. In addition, we want the λ parameters to be constructed such that that the scaling (28) for *ε*_CaL_, *ε*_NaL_, and *ε*_Kr_ is sufficient to fit the model to the drug doses under consideration. In order to increase the chance of obtaining such a control model, we fit the control parameters, λ, and the drug parameters, *ε*, simultaneously, instead of first finding the optimal control parameters, λ, by fitting the base model to the control data, and then subsequently finding appropriate drug parameters, *ε*, for each dose. In addition, all doses are included in the inversion, so that the estimated values of *ε* are based on *all* the drug doses included in the data set.

In order to illustrate the role of the λ- and *ε*-parameters more clearly, consider a simplified model consisting of just two currents, and assume that the base model is given by (see the section *Modeling the Membrane Currents*)

(29) $$\frac{dv}{dt}=-g_{1}o_{1}(v-E_{1})-g_{2}o_{2}(v-E_{2}).$$

Assume further that we have data from cells with both no drug present and with different doses of a drug (e.g., one low dose and one high dose). We assume that the drug may block any of the two model currents. In the inversion procedure, we try to find optimal values of the four parameters λ_1_, λ_2_, *ε*_1_, and *ε*_2_ so that the adjusted model of the form

(30) $$\frac{dv}{dt}=-\frac{1+\lambda_{1}}{1+D\varepsilon_{1}}g_{1}o_{1}(v-E_{1})-\frac{1+\lambda_{2}}{1+D\varepsilon_{2}}g_{2}o_{2}(v-E_{2})$$

fits the data both for the control case (*D* = 0) and for the considered drug doses. In other words, for a given parameter set λ_1_, λ_2_, ε_1_, and ε_2_, we need to compute the solution of the model (30) both for the control case (*D* = 0) and for the considered drug doses and compare the obtained solutions to the corresponding experimental data.

The more general case considered in our computations is conceptually identical; however, as we also consider scaling of parameters that are not assumed to possibly be affected by the drug, we also have some parameters simply scaled by a factor (1+λ*_i_*) instead of by $\frac{1+\lambda_{i}}{1+D\varepsilon_{i}}$.

#### Properties of the Cost Function

In order to find the optimal parameters for fitting the model to data, we need to define a cost function that measures the difference between a given model solution and the data. This cost function is defined as

(31) $$H(\lambda ,\varepsilon )=\sum_{d}\sum_{j}w_{d,j}{\left(H_{j}(\lambda ,\varepsilon ,D_{d})\right)}^{2}.$$

Here, *d* runs over each of the considered drug doses, *D_d_*, including the control case (D_0_ = 0), and *j* runs over each cost function term, *H_j_*, representing various differences between the data and the model solution. The parameters *w_d,j_* represent weights for each of the cost function terms for each of the doses. Each of the cost function terms, *H_j_*, are defined in Section S3.1 of the **Supplementary Information**.

#### A Continuation-Based Minimization Method

As outlined above, we wish to adjust the base model to data by finding λ- and ε-parameters that minimize a cost function of the form (31), measuring the difference between the input data and the model solution. In order to search for the optimal values of λ and ε, we apply a continuation-based optimization method [see e.g., (Keller, 1987; Allgower and Georg, 2012)]. Briefly, continuation is used to simplify the solution of equations or of optimization problems by introducing a *θ*-parameterization such that the solution is known for one value of θ. Suppose, for instance, that the parameterization is defined such that the solution is known for θ = 0 and the problem we want to solve is defined by *θ* = 1. Then the solution at *θ* = 1 can be found by starting at *θ* = 0 and carefully step towards the solution at θ = 1. One advantage with this method is that we can start at a solution that we know is correct (at *θ* = 0) and then take *small steps* towards the goal at *θ* = 1. For the problem of inverting membrane potential and Ca^2+^ traces, this method has proven to be useful.

##### Cost function in the continuation case

More specifically, we assume that, for each drug dose, *D_d_*, (including the control case) the data we are trying to invert are given by some vector pair [*v*^1^(*D_d_*), *c*^1^(*D_d_*)], where *v*^1^(*D_d_*) is the membrane potential and *c*^1^(*D_d_*) is the Ca^2+^ concentration. In addition, from the default base model specified by λ = ε = 0, we can compute a vector pair (*v*^0^, *c*^0^) for the membrane potential and Ca^2+^ concentration as the starting point of the inversion.

The goal of the continuation method is to compute a path for λ and ε from λ = *ε* = 0, which fit (*v*^0^, *c*^0^) perfectly, to some λ and *ε* that fit the final data [*v*^1^(*D_d_*), *c*^1^(*D_d_*)] for each of the drug doses, *D_d_*, as best as possible. This is done by defining a cost function of the form

(32) $$\bar{H}(\theta ,\lambda ,\varepsilon )=\sum_{d}\sum_{j}w_{d,j}{\left({\bar{H}}_{j}(\theta ,\lambda ,\varepsilon ,D_{d})\right)}^{2},$$

for the intermediate steps in the algorithm. Here, θ is a parameter that is gradually increased from zero to one. In the definition (32), the terms ${\bar{H}}_{j}(\theta ,\lambda ,\varepsilon ,D_{d})$ correspond to each of the terms *H_j_*(λ, ε, *D_d_*) defined in Section S3.1 of the **Supplementary Information**. Specifically, the terms take the form

(33) $${\bar{H}}_{j}(\theta ,\lambda ,\varepsilon ,D_{d})=\frac{\left|R_{j}(v(\lambda ,\varepsilon ,D_{d}),c(\lambda ,\varepsilon ,D_{d}))-R_{j}^{\theta }(D_{d})\right|}{\left|R_{j}^{\theta }(D_{d})\right|},$$

(34) $$R_{j}^{\theta }(D_{d})=(1-\theta )R_{j}(v^{0},c^{0})+\theta R_{j}(v^{1}(D_{d}),c^{1}(D_{d})),$$

where *R_j_*(*v*,*c*) represent different characteristics of the AP or Ca^2+^ transient, e.g., the AP duration at some percentage or the upstroke velocity (see Section S3.1 of the **Supplementary Information** ^1^). In the case $\theta =0,{\text{ R}}_{j}^{\theta }(D_{d})$ is equal to the terms defined by the default model (λ = ε = 0) for all the doses *D_d_*. Therefore, $\bar{H}(0,0,0,D_{d})=0,$ ^2^ so the optimal solution for θ = 0 is λ = ε = 0. In the case θ = 1, the terms ${\text{R}}_{j}^{\theta }(D_{d})$ are equal to the characteristics computed for the data we wish to invert. In other words, $\bar{H}(1,\lambda ,\varepsilon )=H(\lambda ,\varepsilon )$, where *H*(λ, ε) is defined in (31). For the intermediate values of θ, the characteristics ${\text{R}}_{j}^{\theta }(D_{d})$ represent weighted averages of characteristics of the model used as a staring point for the inversion (λ = ε = 0) and the data we are trying to invert. Therefore, we expect the optimal values of λ and ε to gradually move from zero to the optimal values for the data as θ is increased from zero to one.

##### The minimization algorithm

In the minimization algorithm, we find the optimal solution in *M* iterations. We define θ*_m_* = Δθ·*m* for *m* = 0,…,*M*, where Δθ = 1/*M*. For *m* = 1,…,*M*, we assume that the optimal values λ(θ*_m_*_˗1_) and ε(θ*_m_*_˗1_) have been computed, and we want to find λ(θ*_m_*) and ε(θ*_m_*) by finding the minimum of $\bar{H}(\theta_{m},\lambda ,\varepsilon )$. Since the step in θ is small, we assume that the changes in λ and ε are also relatively small. We use the Nelder-Mead algorithm (Nelder and Mead, 1965) to minimize $\bar{H}(\theta_{m},\lambda ,\varepsilon )$, and we use λ(θ*_m_*_˗1_) and ε(θ*_m_*_-1_) as suggestions for the starting vectors to find λ(θ*_m_*) and ε(θ*_m_*). However, in order to increase the chance of finding the true optimal value in every iteration, we start the Nelder-Mead algorithm from several randomly chosen starting vectors in the vicinity of λ(θ*_m_*_˗1_) and ε(θ*_m_*_˗1_). **Figure S3** in the **Supplementary Information** illustrates the development of the ε-values in an inversion aiming to characterize a drug.

##### Technical specifications

In the applications presented below, we use *M* = 20, and in each iteration *m*, we draw 63 guesses (as the specific computer used for these simulations has 64 cores) for the starting vectors for the Nelder-Mead algorithm from [λ(θ*_m_*_-1_)−0.2, λ(θ*_m_*_-1_)+0.2] and $\left[\frac{\varepsilon (\theta_{m-1})}{5},5\cdot \varepsilon (\theta_{m-1})\right]$ for λ and ε, respectively. In the first 15 iterations, we use five iterations of the Nelder-Mead algorithm for each guess, and for the last five iterations we use 25 iterations of the Nelder-Mead algorithm. For each new parameter set, we generally run the simulation for 15 AP cycles using 1 Hz pacing before measuring the AP and Ca^2+^ transient, unless otherwise specified. The choice of 15 AP cycles is selected as a compromise between the desire of minimizing the computational efforts required for each cost function evaluation and the desire of reaching new stable steady state values for the state variables following a parameter change. Presently, each cost function evaluation requires about 14 sec of computing time for a data set including four doses in addition to the control case.

### Identifiability of the Base Model Based on Singular Value Decomposition of Currents

In the inversion procedure outlined above, we try to find the optimal adjustment factors λ and ε for the model so that the AP and cytosolic Ca^2+^ transient in the model solution match measurements of the AP and Ca^2+^ transient as best possible. An important element to consider in this process is whether the identified adjustment factors found by the inversion procedure are the only combination of adjustment factors that fit the data, or whether other adjustment factors might exist which fit the data equally well.

In order to investigate the identifiability of the adjustment factors for the currents in the base model, we apply a method based on singular value decomposition [see, e.g., (Liesen and Mehrmann, 2015; Lyche, 2017)] of the currents. This approach is described in detail in (Jæger et al., 2019a). In short, the identifiability of the currents is investigated by collecting the model currents at time points *t_n_ = n*Δ*t*, for *n* = 1, …, *N_t_* into a matrix $A\in R^{N_{t},N_{c}}$, where *N_c_* is the number of model currents. Then, the singular value decomposition of the matrix

$$A=USV^{T}$$

is computed. Here, the matrices $U\in R^{N_{t},N_{t}}$ and $V\in R^{N_{c},N_{c}}$ are unitary matrices, and the matrix $S\in R^{N_{t},N_{c}}$ is a diagonal matrix with singular values *σ_i_* along the diagonal. The columns *u_i_* and *v_i_* of *U* and *V*, respectively, are the associated singular vectors.

From the properties of the singular value decomposition it can be shown that perturbations of the adjustment factors along singular vectors *v_i_* associated with large singular values *σ_i_* are expected to result in significant changes in the AP, whereas perturbations of the adjustment factors along singular vectors *v_i_* associated with small singular values are, accordingly, expected to result in small changes in the AP.

In (Jæger et al., 2019a) it was shown that this expected result seemed to hold in the case of three well-known AP models of adult ventricular cardiomyocytes. In addition, it was demonstrated how this analysis could be used to define an *identifiability index* for individual model currents. This index was defined for each current *j*=1,...,*N_c_* as

(35) $$k(e_{j})={\left||e_{j}-P_{N}e_{j}|\right|}_{2},$$

where $e_{j}\in R^{N_{c}}$ is the vector that is one in element number *j* and zero elsewhere. Moreover, $P_{N}e_{j}\in R^{N_{c}}$ is the projection of *e_j_* onto the unidentifiable space spanned by the singular vectors *v_i_* associated with small singular values (or small perturbation effects). In other words, if *k*(*e_j_*) is close to zero, almost the entire current *I_j_* is in the unidentifiable space, and we cannot be sure that the value of the associated adjustment factors λ*_j_* or ε*_j_* are the only values that fit the data (i.e., result in the same AP). On the other hand, if *k*(*e_j_*) is close to one, we expect that other values of λ*_j_* or ε*_j_* would not fit the data as well as the currently assumed values, as perturbations of the adjustment factors would result in large changes in the AP.

Note that this approach only aims to determine the identifiability of the adjustment factors for the membrane currents. The analysis could be extended to include other state variables than just the membrane potential (e.g., the Ca^2+^ concentrations). In this case, the identifiability of the remaining adjustment factors might also be suggested. However, at this stage primary focus is on identifying drug effects on membrane ion channels, so we are principally interested in ensuring that the adjustment factors for the currents are unique.

## Results

Below, we demonstrate a few applications of the method outlined above. First, in the section *The Base Model*, we define the default hiPSC-CM and adult parameterizations of the general base model formulation. We also demonstrate that these models exhibit high gain and graded release of the Ca^2+^ fluxes. In addition, we illustrate the identifiability of the model currents using SVD analysis, as described above. This analysis is used to determine which model currents should be fixed in the applications of the inversion procedure. Next, in the section *Identification of Drug Effects on hiPSC-CMs Based on Simulated Data*, we use the inversion procedure to identify drug effects for data generated by simulations. Finally, in the section *Identification of Drug Effects on hiPSC-CMs Based on Optical Measurements*, we apply the inversion procedure to identify drug effects from data obtained from optical measurements of hiPSC-CMs.

### The Base Model

Here, we set up the default adult and hiPSC-CM base model formulations used in the inversion procedure in the following sections.

#### Base Model Approximation of the Grandi Model

**Figure 3:**
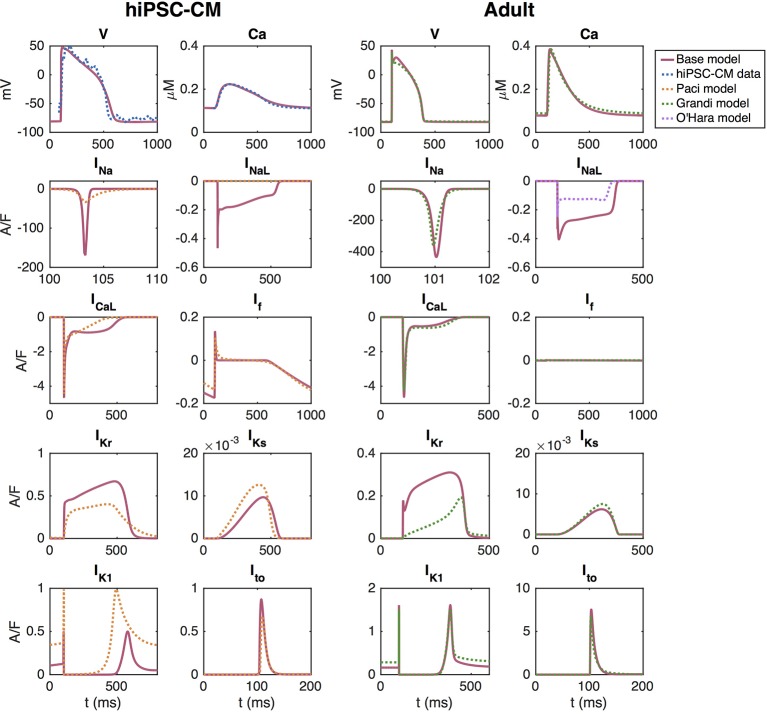
AP, cytosolic Ca^2+^ transient and major ionic currents in the hiPSC-CM and adult versions of the base model. In the left panel, the base model is adjusted to fit data obtained from optical measurements of the AP and Ca^2+^ transient of hiPSC-CMs. In the right panel, the base model is adjusted to approximate the Grandi et al. (2010) model of adult cardiomyocytes.

The adult base model is fitted to approximate the Grandi et al. model using the inversion procedure described above. The upper right panel of **Figure 3** shows the AP and Ca^2+^ transient of the Grandi et al. (2010) model for healthy adult ventricular cardiomyocytes and the AP and Ca^2+^ transient of the adult version of the base model. In the lower right panels, we compare a number of major ionic currents in the base model to those in the Grandi et al. (2010) or the O’Hara et al. (2011) models.

In the inversion, the cost function includes all the terms specified in Section S3.1 of the **Supplementary Information**, except for the regularization terms. For the cost function terms involving information about currents and fluxes, we have included *I*_Na_, *I*_CaL_, *I*_to_, *I*_Kr_, *I*_Ks_, *I*_K1_, *I*_NaCa_, *I*_pCa_, and *I*_bCa_, as well as the fluxes *J*_RyR_ and *J*_SERCA_ (see Section S3.1.7 of the **Supplementary Information**). All terms measuring the difference in membrane potential or Ca^2+^ concentration are given the weight *w_j_* = 1 and the terms measuring differences in the currents are given the weight *w_j_* = 0.5. The initial conditions for the parameters included in the inversion are specified in **Table S9** of the **Supplementary Information**.

As mentioned above, we define the *default* base model as the adult base model because this model will remain fixed, whereas the hiPSC-CM models will change depending on experimental data. The parameter values obtained in the inversion procedure therefore define the default base model and are specified in Section S1 of the **Supplementary Information**.

#### Default Base Model for hiPSC-CMs

The left panel of **Figure 3** shows the solution of the default base model for hiPSC-CMs fitted to optical measurements of the AP and Ca^2+^ transient. In this case, the cost function consists of the terms *H*_APD30_, *H*_APD50_, *H*_APD80_, *H*_CaD20_-*H*_CaD80_, *H*_int30_, *H*_dvdt_, *H*_dcdt_, *H*_Ca_, *H*_Ca,b_, $H_{I_{\mathrm{Na}}^{\max}}$, $H_{I_{\mathrm{CaL}}^{\max}}$, $H_{I_{\mathrm{Kr}}^{\max}}$, $H_{I_{\mathrm{Ks}}^{\max}}$, $H_{I_{\text{K}1}^{\max}}$, $H_{I_{\mathrm{to}}^{\max}}$, $H_{I_{f}^{\max}}$ (see the **Supplementary Information**), where the information about the currents is obtained from the Paci et al. model (Paci et al., 2013) which is based on patch-clamp recordings of the ionic currents of hiPSC-CMs from (Ma et al., 2011). The terms *H*_CaD20_-*H*_CaD75_ are given the weight 0.5, and *H*_APD80_ and *H*_CaD80_ are given the weight 5. Furthermore, $H_{I_{\mathrm{Na}}^{\max}}$, $H_{I_{\mathrm{Ks}}^{\max}}$, $H_{I_{\text{K}1}^{\max}}$, $H_{I_{\mathrm{to}}^{\max}}$, and $H_{I_{f}^{\max}}$ are given the weight 0.5 and $H_{I_{\mathrm{CaL}}^{\max}}$ and $H_{I_{\mathrm{Kr}}^{\max}}$ are given the weight 5. The remaining terms are given the weight 1.

The mapping between hiPSC-CMs and adult cardiomyocytes returned by the inversion procedure are reported in **Table 1**. Note that these factors represent the default hiPSC-CM base model to be used as a starting point for the inversion of the remaining control data sets. In other words, the specific adjustment factors between the hiPSC-CM and adult models will differ for each new data set. Note also that in our applications of the inversion procedure, we consider data from stimulated hiPSC-CMs, and therefore, the hiPSC-CMs are not required to be spontaneously beating. The default hiPSC-CM version of the base model in **Figure 3** is not spontaneously beating, and whether the model fitted to a specific data set is spontaneously beating or not will depend on the fitted parameter values. In **Figure S4** in the Supplementary material, we report how some of the AP and Ca^2+^ transient features depend on the pacing frequency for the default hiPSC-CM and adult base models and compare the results to those of the Grandi et al. (2010), O’Hara et al. (2011), Paci et al. (2013), and Kernik et al. (2019) models for adult and hiPSC-derived cardiomyocytes.

**Table 1:** Values defining the maturation map between the default hiPSC-CM and adult base models illustrated in **Figure 3**.

λ_Na_	2.00	λ_NaK_	-0.16	$\lambda_{sl}^{c}$	–0.14
λ_NaL_	-0.08	λ_CaL_	-0.53	$\lambda_{n}^{s}$	–0.10
λ_Kr_	-0.54	λ_bCa_	3.90	$\lambda_{d}^{c}$	–0.61
λ_Ks_	0.68	λ_pCa_	-0.85	$\lambda_{B}^{c}$	–0.56
λ_K1_	2.23	λ_NaCa_	-0.69	$\lambda_{B}^{d}$	–0.72
λ_to_	8.45	λ_RyR_	-0.20	$\lambda_{B}^{sl}$	–0.60
λ_f_	-0.99	λ_SERCA_	-0.53	$\lambda_{B}^{s}$	–0.58
λ_bCl_	42.43	λ_χ_	-0.33		

The adult parameters, *p*^A^, are related to the hiPSC-CM parameters, *p*^hiPSC^, by the relation *p*^A^ = (1+λ)*p*^hiPSC^. See the sections *Modeling the Membrane Currents*, *Modeling Intracellular Ca^2+^ Dynamics* and Section S2.1 in the **Supplementary Information** for more detailed definitions of each of the λ-values.

#### High Gain and Graded Release of the Base Model

As mentioned above, the base model formulation of Ca^2+^ release is designed to exhibit both high gain and graded release. This has proved impossible to achieve using common pool models [see, e.g., (Rice et al., 1999; Dupont et al., 2016)], as discussed in more detail in the **Supplementary Information**. The Ca^2+^ release model we have designed differs from the classical common pool models in two ways: first, release of Ca^2+^ from the SR is not directed into the dyad (d), but rather directly to the subsarcolemmal (SL) space (see **Figure 2**), and, second; the release mechanism is formulated in terms of both an availability rate and open probability for the RyRs [see (16)].

**Figure 4:**
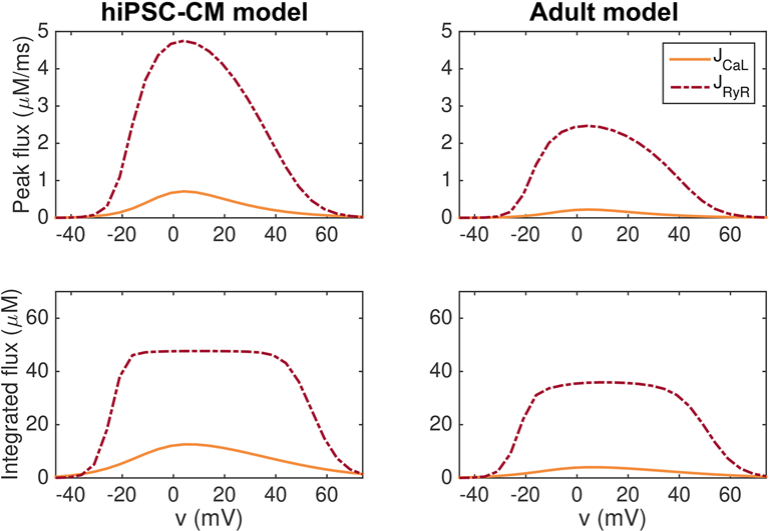
Graded release for the hiPSC-CM (left) and adult (right) versions of the base model. In the upper panel, we report the peak of the *J*_CaL_ and *J*_RyR_ fluxes for simulations in which the membrane potential is fixed at specific values between −50 mV and 80 mV. In the lower panel, we show the fluxes integrated with respect to time from *t* = 0 ms to *t* = 100 ms. After 100 ms, both *J*_CaL_ and *J*_RyR_ have roughly returned to their resting levels.

In **Figure 4**, we show that this model exhibits high gain and graded release both when the hiPSC-CM and adult parameters are applied. In the figure, we report the peak of the *J*_CaL_ and *J*_RyR_ fluxes as well as the integrated fluxes for simulations in which the membrane potential is fixed at specific values. The remaining state variables of the model start at the default initial conditions corresponding to the default resting membrane potential of the model, and the simulations record the *J*_CaL_ and *J*_RyR_ fluxes resulting from the clamped membrane potential.

We observe that for most values of *v*, the *J*_RyR_ flux is considerably larger than the *J*_CaL_ flux, indicating high gain. Furthermore, a small *J*_CaL_ flux seems to be associated with a small *J*_RyR_ flux, whereas a large *J*_CaL_ flux is associated with a large *J*_RyR_ flux, indicating graded release.

#### Identifiability of the Currents in the hiPSC-CM Base Model

In order to investigate the identifiability of the individual model currents, we apply the singular value decomposition analysis from (Jæger et al., 2019a) described in the section *Identifiability of the Base Model Based on Singular Value Decomposition of Currents*.

**Figure 5:**
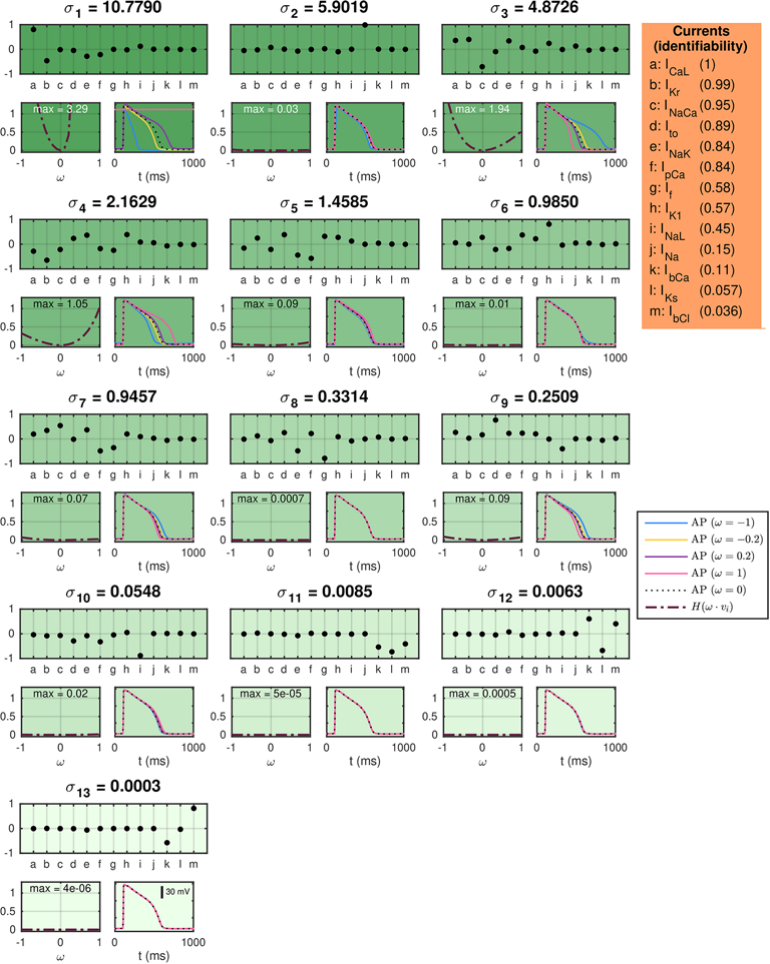
SVD analysis of the currents in the default base model for hiPSC-CMs. The titles above each plot give the singular values of the current matrix *A*, and the upper plots show the corresponding singular vectors. The below plots show how a perturbation of the currents corresponding to the singular vector affects the computed AP for a few examples (right) and measured by a cost function (left). The identifiability index (35) of each current is given in the orange panel.

In **Figure 5**, titles above each plot indicate the value of each of the singular values of the current matrix, *A*. The upper plots below the singular values show the singular vectors corresponding to each of the singular values. Here, each letter corresponds to a single current specified in the table on the right-hand side. The below left plots show the values of the cost function (31) evaluated using the default base model for hiPSC-CMs as data and a perturbed model as the model solution. In the perturbed model, the maximum conductances are perturbed with λ-values [see (5)] equal to *ω* ***∙****v_i_*, where *v_i_* is the considered singular vector and *ω* is varied between zero and one. The cost function includes the terms *H*_APD30_, *H*_APD50_, *H*_APD80_, and *H*_Int30_ with weight 1 for all terms except *H*_APD80_, which is given the weight 5 (see the **Supplementary Information** for definitions). The maximum values of *H* are given in the top of the plots. The right plots show the solutions resulting from the perturbations for a few selections of *ω*.

In (Jæger et al., 2019a) it was shown that perturbations of the maximum conductances along singular vectors corresponding to large singular values generally resulted in large perturbation effects, whereas perturbations along singular vectors corresponding to small singular values generally resulted in small perturbation effects for the Ten Tusscher and Panfilov, (2006), the Grandi et al. (2010) and the O’Hara et al. (2011) AP models. **Figure 5** shows correspondence to this result for the hiPSC-CM base model; the main discrepancy is observed for *σ*_2_, which corresponds to a singular vector consisting almost exclusively of the fast sodium current, *I*_Na_. The perturbation effects may be very small for this singular value because the upstroke velocity, physiologically governed by *I*_Na_, is not included in the cost function [cf. (Jæger et al., 2019a)].

In order to quantify the identifiability of the individual currents, we compute the identifiability index, *k*, defined in (35). The unidentifiable space is defined as the space spanned by the singular vectors *v_i_* whose maximum value of H(*ω*·*v_i_*) for 0≤*ω*≤1 is smaller than 0.05. The computed values of the identifiability index for each of the model currents are given in the orange box on the right-hand side of **Figure 5**. A value of *k* close to 1 indicates a high degree of identifiability, while a value of *k* close to 0 indicates an unidentifiable current.

From the indices in **Figure 5**, we see that *I*_CaL_, *I*_Kr_, and *I*_NaCa_ are highly identifiable, but that the currents *I*_NaL_, *I*_Na_, *I*_bCa_, *I*_Ks_, and *I*_bCl_ has an identifiability index below 0.5. As a consequence, we fix the conductance of *I*_Na_, *I*_bCa_, *I*_Ks_, and *I*_bCl_ in the applications of the inversion procedure presented below. In addition, we are aware that the *I*_NaL_ current might be hard to identify, and that estimated drug effects for this current are associated with a level of uncertainty [see also (Poulet et al., 2015)].

### Identification of Drug Effects on hiPSC-CMs Based on Simulated Data

Our first application of the inversion procedure is to identify drug effects as based on simulated data. To generate these data, we set λ_CaL_ = λ_NaL_ = λ_Kr_ = 0.1 in the default hiPSC-CM base model. In addition, we apply a set of *ε*-values to represent five specific drugs—Nifedipine, Lidocaine, Cisapride, Flecainide, and Verapamil. We assume that Nifedipine is a pure *I*_CaL_-blocker with an IC50 value of 10 nM, that Lidocaine is a pure *I*_NaL_-blocker with an IC50 value of 10 *μ*M, and that Cisapride is a pure *I*_Kr_-blocker with an IC50 value of 10 nM. Furthermore, Flecainide is assumed to block a combination of all three currents with IC50 values of 25 *μ*M, 20 *μ*M and 10 *μ*M for *I*_CaL_, *I*_NaL_, and *I*_Kr_, respectively. Verapamil is assumed to block *I*_CaL_ with an IC50 value of 200 nM and *I*_Kr_ with an IC50 value of 500 nM. Both when the data is generated and in the inversion procedure, we record the sixth generated AP following each parameter change.

**Figure 6:**
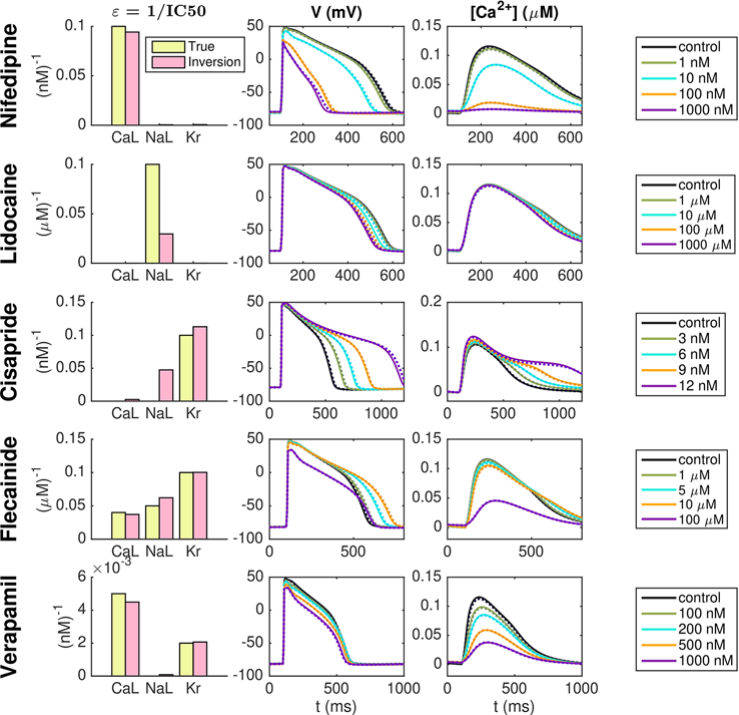
Identification of drug effects for five drugs based on simulated data. The λ-values λ_CaL_, λ_NaL_, and λ_Kr_ and the *ε*-values *ε*_CaL_, *ε*_NaL_, and *ε*_Kr_ are allowed to vary in the inversion. The left panel shows the *ε*-values used to generate the simulated drug data (yellow) and the corresponding *ε*-values estimated by the inversion procedure (pink). The center and right panels show the AP and Ca^2+^ transients, respectively, for each of the drug doses included in the data sets. Solid lines represent the simulated data and dotted lines show the fitted model solutions returned by the inversion procedure. Note that to clearly see changes in the Ca^2+^ transient amplitude, the Ca^2+^ transients are adjusted so that the Ca^2+^ transient amplitude is preserved, but the minimum value is set to zero in all cases.

**Figure 6** shows the result of the inversion procedure using the λ-values λ_CaL_, λ_NaL_, and λ_Kr_ and the *ε*-values *ε*_CaL_, *ε*_NaL_, and *ε*_Kr_ as free parameters in the inversion procedure. The left panel shows the *ε*-values used to generate the data (yellow) and the corresponding *ε*-values returned by the inversion procedure (pink). The center and right panels show the AP and Ca^2+^ transient, respectively, for the control case and for each of the drug doses included in the data sets. The solid lines show the simulated data and the dotted lines show the solutions generated by the model using the λ- and *ε*-values returned by the inversion procedure. Note that to clearly see differences in the Ca^2+^ transient amplitude, the Ca^2+^ transients are adjusted so that the Ca^2+^ transient amplitude is preserved, but the minimum Ca^2+^ concentration is set to zero. We observe that the inversion procedure is able to identify the correct *ε*-values accurately, excepting the *ε*-value for Lidocaine, which is predicted to be considerably lower than the value used to generate the data.

### Identification of Drug Effects on hiPSC-CMs Based on Optical Measurements

Below, we present use of the inversion procedure to identify drug effects on hiPSC-CMs from optical measurements of the AP and Ca^2+^ transient.

#### Nifedipine

**Figure 7:**
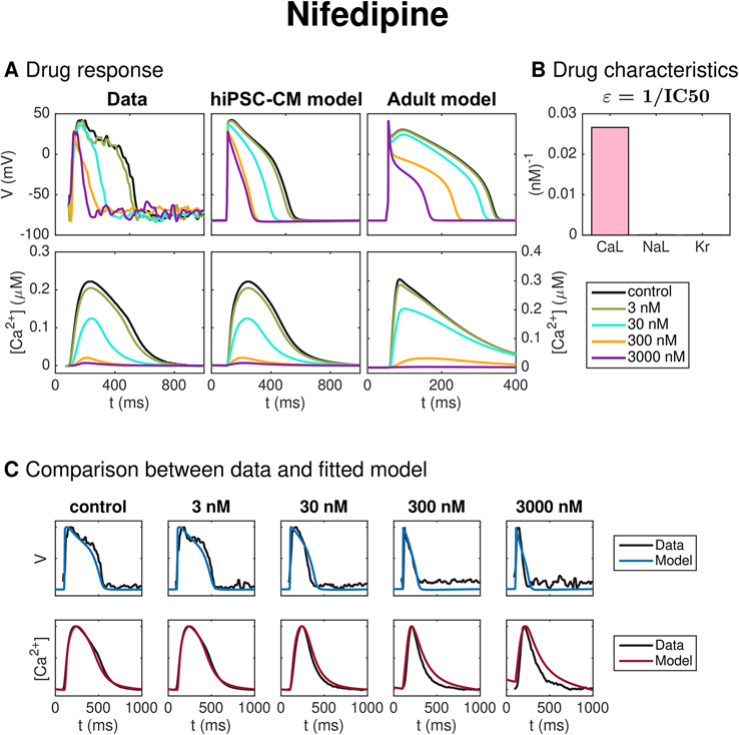
Identification and mapping of drug effects for the drug Nifedipine based on optical measurements of the AP and Ca^2+^ transient of hiPSC-CMs. **(A)** AP and Ca^2+^ transient in the control case and for four drug doses for the data (left) and the fitted hiPSC-CM model (center). The predicted drug effects for adult cardiomyocytes are given in the right panel (note that the scaling of the axes is adjusted for the adult case). Note also that, to clearly see differences in the Ca^2+^ transient amplitude, the displayed Ca^2+^ concentrations are adjusted so that the Ca^2+^ transient amplitude is preserved, but the resting concentration is set to zero in each case. **(B)** Drug effect on *I*_CaL_, *I*_NaL_ and *I*_Kr_ in the form of *ε*-values estimated by the inversion procedure. **(C)** Comparison between the measured membrane potential and Ca^2+^ traces and the fitted model solutions for each of the doses in the data set.

**Figure 7** shows the result of the inversion procedure applied to data from optical measurements of hiPSC-CMs exposed to the drug Nifedipine. The data includes the control case with no drug present and four different drug doses (3 nM, 30 nM, 300 nM, and 3,000 nM). The left panel of **Figure 7A** shows the membrane potential and Ca^2+^ traces obtained from optical measurements, and the center panel shows the corresponding solutions of the hiPSC-CM version of the base model fitted to the optical measurements. Note that the values of the data are mapped so that the maximum and minimum values of the membrane potential and Ca^2+^ concentration match those of the fitted hiPSC-CM model. Panel C of **Figure 7** compares the experimentally measured data and the fitted model for each of the doses. We observe that the model seems to fit the data quite well for most of the doses, but that the Ca^2+^ transient appears to last a bit longer in the model than in the data for the highest considered drug doses.

The dose-dependent effect of the drug on the *I*_CaL_, *I*_NaL_, and *I*_Kr_ currents are modeled using IC50 values (see Section *IC50 Modeling of Drug Effects*). The values of $\varepsilon_{i}=\frac{1}{IC50_{i}}$ for *i* = CaL, NaL, and Kr are given in **Figure 7B**. A large value of ε*_i_* corresponds to a large drug effect on the current *i*, and a small value of ε*_i_* corresponds to a small drug effect on the current *i*. From **Figure 7B**, we observe that the inversion procedure predicts that Nifedipine primarily blocks *I*_CaL_.

The IC50 values corresponding to the estimated *ε*-values for *I*_CaL_, *I*_NaL_ and *I*_Kr_ are given and compared to literature values in **Table 2** for all the five considered drugs of this section (see the *Discussion* section for a discussion of these results).

**Table 2:** Comparison between the IC50 values obtained from the inversion procedure and values found in literature.

		Nifedipine	Lidocaine	Cisapride	Flecainide	Verapamil
CaL	**Inversion**	**38 nM**	**3400 *μ*M**	**775 nM**	**9 *μ*M**	**495 nM**
	Literature	12 nM (Kramer et al., 2013)		11 800 nM (Kramer et al., 2013)	26 *μ*M (Crumb et al., 2016)	202 nM (Crumb et al., 2016)
		60 nM (Di Stilo et al., 1998)			27 *μ*M (Kramer et al., 2013)	200 nM (Kramer et al., 2013)
						100 nM (Mirams et al., 2011)
NaL	**Inversion**	**23 600 nM**	**4.3 *μ*M**	**120 nM**	**47 *μ*M**	**23 000 nM**
	Literature		11 *μ*M (Crumb et al., 2016)		19 *μ*M (Crumb et al., 2016)	
Kr	**Inversion**	**40 200 nM**	**50 000 *μ*M**	**13 nM**	**1.9 *μ*M**	**2150 nM**
	Literature	440 000 nM (Kramer et al., 2013)		12 nM (Crumb et al., 2016)	0.7 *μ*M (Crumb et al., 2016)	499 nM (Crumb et al., 2016)
		275 000 nM (Zhabyeyev et al., 2013)		20 nM (Kramer et al., 2013)	1.5 *μ*M (Kramer et al., 2013)	250 nM (Kramer et al., 2013)
				6.5 nM (Mohammad et al., 1997)		143 nM (Zhang et al., 1999)

#### Lidocaine

**Figure 8:**
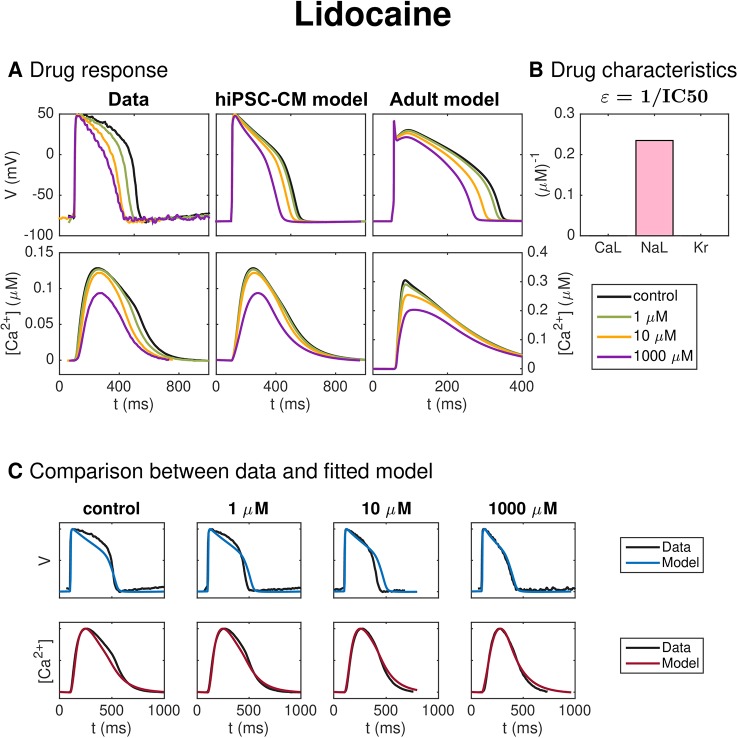
Identification and mapping of drug effects for the drug Lidocaine based on optical measurements of the AP and Ca^2+^ transient of hiPSC-CMs following the same structure as **Figure 7**.

**Figure 8** shows similar results for inversion of measurements of hiPSC-CMs exposed to the drug Lidocaine. In panel A, we observe that the AP duration is reduced by the drug, and in panel B, we observe that the inversion procedure predicts that the drug primarily blocks the *I*_NaL_ current.

#### Cisapride

**Figure 9:**
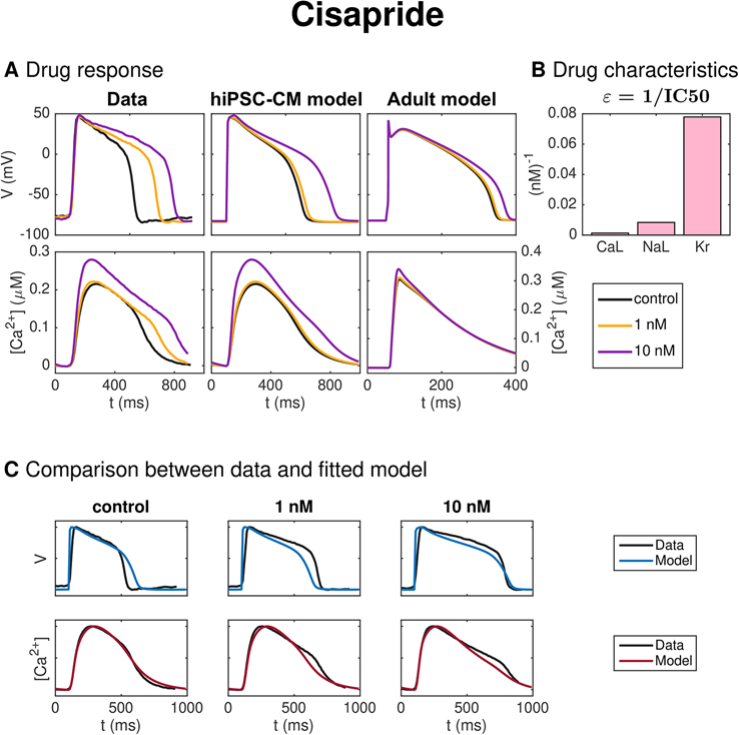
Identification and mapping of drug effects for the drug Cisapride based on optical measurements of the AP and Ca^2+^ transient of hiPSC-CMs following the same structure as **Figure 7**.

**Figure 9** shows the result of the inversion procedure applied to a data set for hiPSC-CMs exposed to the drug Cisapride. In panel A, we observe that the drug increases the AP duration. In panel B, we observe that the inversion procedure predicts that Cisapride primarily blocks the *I*_Kr_ current.

#### Flecainide

**Figure 10:**
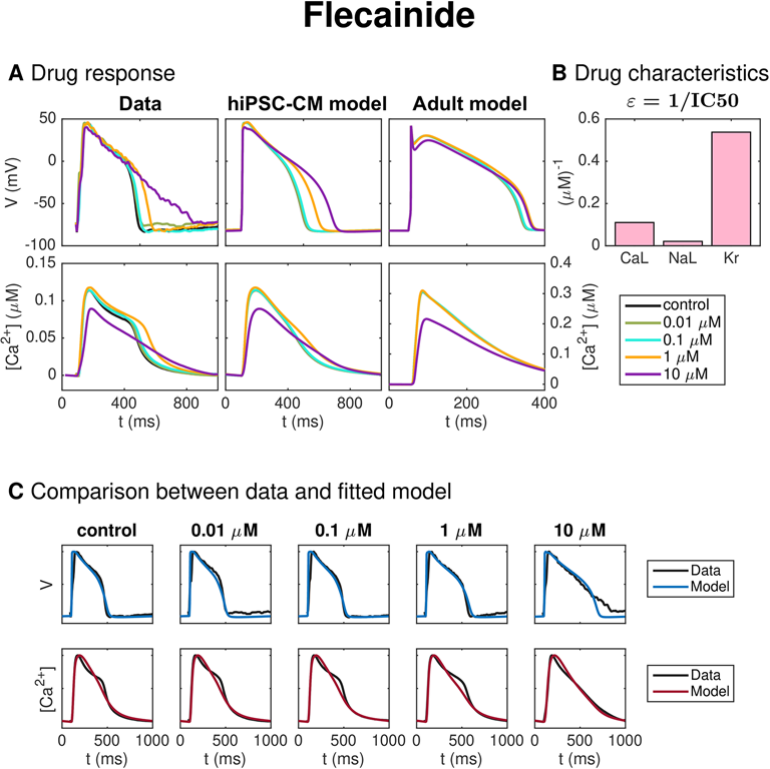
Identification and mapping of drug effects for the drug Flecainide based on optical measurements of the AP and Ca^2+^ transient of hiPSC-CMs following the same structure as **Figure 7**.

**Figure 10** shows the result for the inversion procedure applied to optical measurements of hiPSC-CMs exposed to the drug Flecainide. In panel A, we observe that the drug causes increased AP duration. In panel C, we observe that the fitted model fits the data quite well, excepting that the AP duration at high percentages of repolarization is longer for the data than for the model for the highest considered dose. In addition, the shape of the Ca^2+^ transient for the low doses is not entirely captured in the model. In panel B, we observe that the inversion procedure estimates that the drug primarily blocks *I*_Kr_ and, to some degree, *I*_CaL_.

#### Verapamil

**Figure 11:**
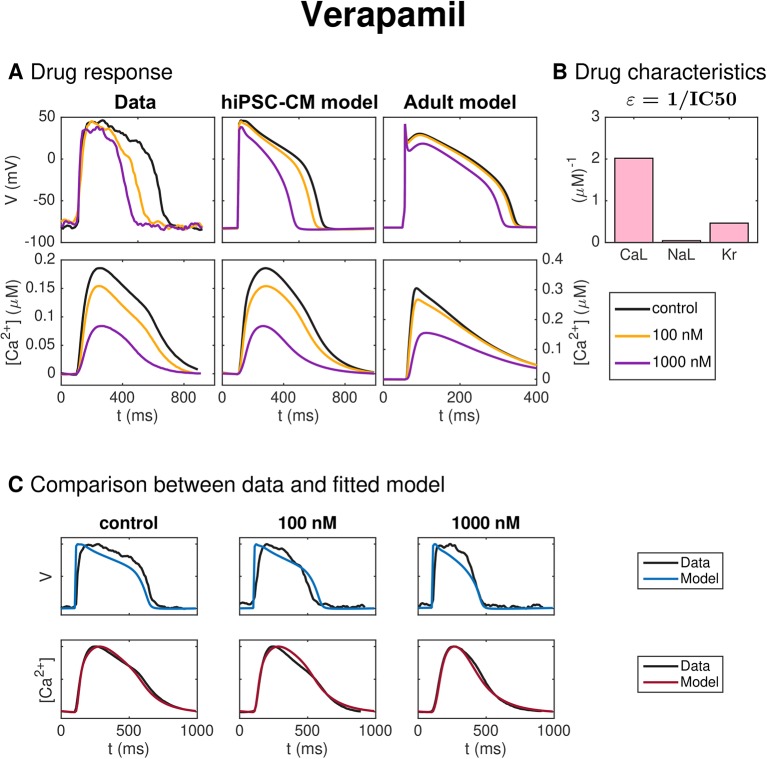
Identification and mapping of drug effects for the drug Verapamil based on optical measurements of the AP and Ca^2+^ transient of hiPSC-CMs, following the same structure as **Figure 7**.

**Figure 11** shows the result of the inversion procedure applied to measurements of hiPSC-CMs exposed to the drug Verapamil. In panel A, we observe that the drug leads to decreased AP duration. Panel B shows that the inversion procedure predicts that Verapamil primarily blocks *I*_CaL_ and, to some extent, *I*_Kr_.

### Mapping of Drug Effects From hiPSC-CMs to Adult Cells

The rightmost plots of panel A of **Figures 7**–**11** show the predicted drug effects for adult cells for each of the drugs considered. More specifically, the plots show the solution of the adult base model exposed to each drug’s effect (*ε*-values) as estimated by the inversion procedure for each of the drug doses included in the data set. To review, this represents the predicted drug response for an adult AP and Ca^2+^ transient exposed to each of the drugs, based on the optical measurements of the AP and Ca^2+^ transient as obtained in a microphysiological system of hiPSC-CMs. The predictions are made by first using the inversion procedure to estimate the effect of the drug on the *I*_CaL_, *I*_NaL_, and *I*_Kr_ currents in the hiPSC-CM case and then mapping the corresponding drug effects to an adult cell using the determined maturation map based on the assumptions of differences in the channel densities and geometry between hiPSC-CMs and adult cardiomyocytes (see the *Methods* section).

## Discussion

Here, we have presented an improved version of the methods presented in (Tveito et al., 2018) for estimating drug effects for adult human cardiomyocytes based on optical measurements of the AP and Ca^2+^ transient of hiPSC-CMs in a microphysiological system. First, we introduce a new base model formulation for representing both adult cells and hiPSC-CMs via different parameter sets. A model for intracellular Ca^2+^ dynamics is updated to a formulation constructed for stability with respect to parameter changes. In addition, we use IC50-based modeling of dose-dependent drug effects and find optimal parameters by running a coupled inversion of both the control data and the drug data for several different doses. The cost function measuring the difference between the data and the model has also been redefined, and we now apply a continuation-based minimization method to minimize the cost function.

### Summary of Method Performance and Main Results: Identification of Drug Effects Based on Simulated Data and Optical Measurements of hiPSC-CMs

**Figure 6** shows the result of the inversion procedure applied to simulated data. As noted above, we observe that the inversion procedure is able to identify the correct *ε*-values accurately, excepting the *ε*-value for Lidocaine, which is predicted to be considerably lower than the value used to generate the data. This suggests that it might be difficult to obtain correct values of ε_NaL_, as also supported by the low identifiability index for *I*_NaL_ reported in **Figure 5**. In addition, we observe that the inversion procedure predicts some block of *I*_NaL_ for the drug Cisapride, even though only *I*_Kr_ was blocked when the data was generated.

We additionally presented the use of the inversion procedure to identify drug effects on hiPSC-CMs from optical measurements of the AP and Ca^2+^ transients.

Panel C of **Figure 7** compares the experimentally measured data and the fitted model for each of the doses of Nifedipine applied to the microphysiological system. The model fits both the membrane potential and the Ca^2+^ data well for most doses applied, although the Ca^2+^ transient duration is longer in the model than in the data for the highest drug doses considered. Furthermore, in panel B, we observe that the inversion procedure predicts that Nifedipine primarily blocks *I*_CaL_. In **Table 2**, we observe that the IC50 value for *I*_CaL_ is estimated to be 38 nM, in agreement with values found in literature (12 nM–60 nM (Di Stilo et al., 1998; Kramer et al., 2013)]. The IC50 value for *I*_NaL_ and *I*_Kr_ are estimated to be 23 600 nM and 40 200 nM, respectively—considerably larger than the doses considered in the data set. We have not found an IC50 value for *I*_NaL_ for comparison in literature, but the IC50 values found for *I*_Kr_ support the claim that the IC50 value is much larger than the drug doses included in the data set, although the literature values [275,000–440,000 nM (Zhabyeyev et al., 2000; Kramer et al., 2013)] are higher than the value predicted by the inversion procedure.

**Figure 8** shows similar results for inversion of measurements of hiPSC-CMs exposed to the drug Lidocaine. The AP duration is reduced by the drug and the inversion procedure predicts that the drug primarily blocks the *I*_NaL_ current. The IC50 value estimate for *I*_NaL_ is 4.3 *μ*M (see **Table 2**), in rough agreement with values found in literature [11 *μ*M (Crumb et al., 2016)]. We observe that the model fits the data quite well, but that the AP duration for the drug dose of 10 *μ*M is longer in the model than in the data.

**Figure 9** shows the result of the inversion procedure applied to a data set for hiPSC-CMs exposed to the drug Cisapride. Considering the leftmost and center panels of **Figure 9A**, we observe that the prolongation of the AP duration is much more prominent in the data as compared to the fitted hiPSC-CM model for a drug dose of 1 nM. This is also confirmed in **Figure 9C**, where we observe that the model does not fit the membrane potential data for the control case and the 1 nM dose case well. The fit for the largest dose, however, is quite good. In **Figure 9B**, we observe that the inversion procedure predicts that Cisapride primarily blocks *I*_Kr_. In **Table 2**, we observe that the IC50 value for *I*_Kr_ is estimated to be 13 nM, in good agreement with values found in literature [6.5 nM–20 nM (Mohammad et al., 1997; Kramer et al., 2013; Crumb et al., 2016)].

**Figure 10** shows the result for the method as applied to measurements of hiPSC-CMs exposed to the drug Flecainide, know to prolong the AP duration. In **Table 2**, we observe that the IC50 value for *I*_Kr_ predicted by the inversion procedure (1.9 *μ*M) is in quite good agreement with literature values [0.7–1.5 *μ*M (Kramer et al., 2013; Crumb et al., 2016)], but that the predicted IC50 value for *I*_CaL_ (9 *μ*M) is too low compared to the reported literature values [26–27 *μ*M (Kramer et al., 2013; Crumb et al., 2016)]. In addition, the estimated IC50 value for *I*_NaL_ (47 *μ*M) is larger than the literature value of 19 *μ*M (Crumb et al., 2016).

In **Figure 11**, the method is applied to measurements of hiPSC-CMs exposed to Verapamil. In panel A, the effect on the AP duration for the smallest dose (100 nM) appears to be more prominent in the data than in the fitted model. This is confirmed in panel C, where we observe that the fitted model seems to fit the Ca^2+^ data considerably better than the membrane potential data. In particular, the AP duration is too short in the control case and too long for the smallest dose of 100 nM. Panel B shows that the inversion procedure predicts that Verapamil primarily blocks *I*_CaL_ and to some extent *I*_Kr_. The predicted IC50 values from **Table 2** (495 nM for *I*_CaL_ and 2150 nM for *I*_Kr_) are both higher than the corresponding values from literature [100–202 nM for *I*_CaL_ (Mirams et al., 2011; Kramer et al., 2013; Crumb et al., 2016)] and 143–499 nM for *I*_Kr_ (Zhang et al., 1999; Kramer et al., 2013; Crumb et al., 2016)].

The rightmost plots of panel A of **Figures 7**–**11** show the predicted drug effects for adult cells for each of the drugs considered. We observe that for some drugs (e.g., Nifedipine and Lidocaine), the drug effects for adult cells are predicted to be approximately as severe as for the hiPSC-CMs. For other drugs, on the other hand, (e.g., Flecainide), the drug effect is predicted to be less severe for the adult cells than for hiPSC-CMs, highlighting the critical importance of maturation phenotype in predictive biophysical models of hiPSC-CMs in pharmacological studies.

### Note on Ongoing Complementary Studies

Other recent work has made strong progress in terms of enabling biophysical modeling approaches to assimilate and otherwise make best use of tailored experimental measurements of hiPSC-CMs. For example, in (Gong and Sobie, 2018), the authors address the need to bridge the gap between the effect of drugs on human adult ventricular cardiomyocytes and the effect on animal or hiPSC experimental models often used in drug screening. This work also successfully generated accurate predictions of the effect of ion channel blocking drugs on human adult ventricular cardiomyocytes as based on simulations of hiPSC-CMs via a regression strategy.

Additional recent studies have advanced specific models and methodological approaches for hiPSC-CMs which incorporate experimental variability from multiple data sources [see, e.g., (Kernik et al., 2019)] with the goal of identifying phenotypic mechanisms and identify key parameter sensitivity. The authors introduce a computational whole-cell electrophysiological model of hiPSC-CMs, composed of single exponential voltage-dependent gating variable rate constants, which are then parameterized to fit experimental measurements of hiPSC-CMs from multiple laboratories (and thus incorporate variability in the single-cell measurements of ionic currents observed experimentally). The authors compare hiPSC-CM and adult cell models to elucidate the primary properties underpinning the phenotype, a mechanistically central goal that was not an aim of our present study.

### Limitations and Notes on Future Work

The model for the intracellular Ca^2+^ dynamics in the base model exhibits both high gain and graded release for the hiPSC-CM and adult parameter sets (see **Figure 4**). However, the assumptions underlying the release model are introduced to obtain a stable model, and not necessarily to represent the underlying physiological mechanisms accurately. Future work necessitates assessment of the Ca^2+^ machinery in the base model and potential redevelopment to more accurately represent physiological Ca^2+^ release from the SR, as relevant.

In addition, the intracellular Na^+^ and K^+^ concentrations are assumed to be constants in the base model formulation. This was done in order to avoid problems with drift of the concentrations following from parameter changes [see, e.g., (Hund et al., 2001; Wilders, 2007; Tveito et al., 2018)]. Moreover, freezing the intracellular Na^+^ and K^+^ concentrations during an action potential had very limited effects of the computed membrane potential and cytosolic Ca^2+^ concentration. However, in some cases, drugs are believed to lead to significant changes in, e.g., the intracellular Na^+^ concentration, which could affect the AP shape [see, e.g., (Brill and Andrew Wasserstrom, 1986; Faber and Rudy, 2000)]. Freezing the Na^+^ and K^+^ concentrations could therefore potentially make the base model less suitable for detecting such drug effects.

Note also that the terms included in the cost function (31) applied in the inversion procedure may in future work be adapted as required by the specific application under consideration. For example, the cost function could be extended to include information about the frequency dependence of important action potential and Ca^2+^ transient features (see **Figure S4** in the **Supplementary Information**) or to include information about the spontaneous activity of the hiPSC-CMs used in the experiments.

In the construction of the maturation map, we currently assume that the function of a single channel is the same for different levels of maturity and that only the geometry of the cell and the number of channels change with maturation. It is, however, perhaps possible for the function of some channels to change during maturation as well. If the conductance of a channel changes during maturation, the same adjustment factors as we have considered may still be applied, but if the dynamics governing the open probability of the channel change, additional adjustment factors would have to be included in the models for the channel open probability.

In addition, we have only looked at a single dose escalation study for each of the drugs investigated, as well as only tested the method on tissues derived from a single stem cell line. Future work will assess the variability of the inversion methodology in combination with noisy and incomplete experimental data obtained through these systems across a range of biological maturation approaches and starting stem cell lines.

Furthermore, we have only considered data of the drug response for hiPSC-CMs in microphysiological systems, and we have not been able to obtain corresponding data for adult human cardiomyocytes. Drug response data for isolated healthy adult human cardiomyocytes are generally very limited for both technical and ethical reasons (Rodriguez et al., 2015; Sala et al., 2017). We have therefore not been able to validate the predicted adult drug effects against experimental data.

Further validation of the methodology will primarily be based on further analysis of optical data. Hopefully, we will be able to perform analysis of many drugs and also include blind testing for drugs with well-known properties. When more data from patch-clamp measurements become available, that will also be very useful for improvements and validation of our methods.

In future work, we will seek to find ways to estimate the sodium current which is not possible to estimate today because of low time resolution in the optical data. Furthermore, we will combine the base model with the bidomain model [see e.g., (Franzone et al., 2014)] to study extracellular waveforms in the chips, and also the more detailed EMI model [see e.g., (Tveito et al., 2017; Jæger et al., 2019b)] where individual cells can be represented. Hopefully, the spatially resolved models can provide improved accuracy of the inversion process as well as test important considerations such as the effect of tissue composition.

## Data Availability Statement

All datasets generated for this study are included in the article/**Supplementary Material**.

## Author Contributions

KJ and AT are responsible for the development of the mathematical framework and computer modeling. VC, BC, and KH are responsible for the generation of data provided from microphysiological systems. HF and SW are responsible for the analysis of data from microphysiological systems. KJ, AT, SW, MM, and HF wrote the manuscript text and created the figures. All authors reviewed the manuscript.

## Funding

We would like to acknowledge the following funding sources: The Research Council of Norway funded INTPART Project 249885, the SUURPh program funded by the Norwegian Ministry of Education and Research, the Peder Sather Center for Advanced Study, NIH-NCATS UH3TR000487, NIH-NHLBI HL130417, and in part by California Institute for Regenerative Medicine DISC2-10090.

## Conflict of Interest

KJ, HF, SW, KH, and AT have financial relationships with Organos Inc, and the company may benefit from commercialization of the results of this research.

The remaining authors declare that the research was conducted in the absence of any commercial or financial relationships that could be construed as a potential conflict of interest.

The reviewer SM declared a shared affiliation, with no collaboration, with the authors, KH, VC, and BC, to the handling editor at time of review.

## Footnotes

1 Note that this does not apply to the regularization terms of the cost function. These terms are assumed to be the same for all values of θ.

2 Note that this relies on either the flux balance term H_Ca,b_ being zero for the default base model or on the weight for this term being zero (see Section S3.1.8 in the Supplementary information).

## References

[B1] Allgower E. L. Georg K. (2012). Numerical continuation methods: an introduction Vol. 13 (Berlin Heidelberg, Germany: Springer Science & Business Media).

[B2] Bedada F. B. Wheelwright M. Metzger J. M. (2016). Maturation status of sarcomere structure and function in human iPSC-derived cardiac myocytes. Biochim. Biophys. Acta (BBA)-Molecular Cell Res. 1863 (7), 1829–1838. 10.1016/j.bbamcr.2015.11.005 PMC4864165 26578113

[B3] Brennan T. Fink M. Rodriguez B. (2009). Multiscale modelling of drug-induced effects on cardiac electrophysiological activity. Eur. J. Pharmaceut. Sci. 36 (1), 62–77. 10.1016/j.ejps.2008.09.013 19061955

[B4] Brill D. M. Andrew Wasserstrom J. (1986). Intracellular sodium and the positive inotropic effect of veratridine and cardiac glycoside in sheep purkinje fibers. Circ. Res. 58 (1), 109–119. 10.1161/01.RES.58.1.109 2417742

[B5] CellML Model Repository (2019). www.cellml.org/models/.

[B6] Chen I. Y. Matsa E. Wu J. C. (2016). Induced pluripotent stem cells: at the heart of cardiovascular precision medicine. Nat. Rev. Cardiol. 13 (6), 333–349. 10.1038/nrcardio.2016.36 27009425 PMC5917945

[B7] Christensen K. Roudnicky F. Patsch C. Burcin. M. (2018). “Requirements for using iPSC-based cell models for assay development in drug discovery,” in Engineering and Application of Pluripotent Stem Cells. Eds. Martin U. Zweigerdt R. Gruh I. (Cham: Springer International Publishing), 207–220. 10.1007/10_2017_23 29071405

[B8] Clancy C. E. Zhu Z. I. Rudy Y. (2007). Pharmacogenetics and anti-arrhythmic drug therapy: A theoretical investigation. AJP: Heart Circulatory Physiol. 292 (1), H66–H75. 10.1152/ajpheart.00312.2006 PMC2034498 16997895

[B9] Crumb W. J. Vicente J. Johannesen L. Strauss D. G. (2016). An evaluation of 30 clinical drugs against the comprehensive *in vitro* proarrhythmia assay (CiPA) proposed ion channel panel. J. Pharmacol. Toxicol. Methods 81, 251–262. Focused Issue on Safety Pharmacology. 10.1016/j.vascn.2016.03.009 27060526

[B10] Davies M. R. Mistry H. B. Hussein L. Pollard C. E. Valentin J.-P. Swinton J. (2012). An in silico canine cardiac midmyocardial action potential duration model as a tool for early drug safety assessment. Am. J. Physiology-Heart Circulatory Physiol 302, H1466–H1480. 10.1152/ajpheart.00808.2011 22198175

[B11] Di Baldassarre A. Cimetta E. Bollini S. Gaggi G. Ghinassi B. (2018). Human-induced pluripotent stem cell technology and cardiomyocyte generation: Progress and clinical applications. Cells 7 (6), 48. 10.3390/cells7060048 PMC6025241 29799480

[B12] Di Stilo A. Visentin S. Cena C. Gasco A. M. Ermondi G. Gasco A. (1998). New 1, 4-dihydropyridines conjugated to furoxanyl moieties, endowed with both nitric oxide-like and calcium channel antagonist vasodilator activities. J. Med. Chem. 41 (27), 5393–5401. 10.1021/jm9803267 9876109

[B13] Dupont G. Falcke M. Kirk V. Sneyd J. (2016). Models of calcium signalling Vol. 43 (Cham, Switzerland: Springer). 10.1007/978-3-319-29647-0

[B14] Edwards A. G. Louch W. E. (2017). Species-dependent mechanisms of cardiac arrhythmia: a cellular focus. Clin. Med. Insights: Cardiol. 11, 1179546816686061. 10.1177/1179546816686061. 28469490 PMC5392019

[B15] Ermentrout G. B. Terman D. H. (2010). Mathematical Foundations of Neuroscience Vol. 35 (New York: Springer-Verlag). 10.1007/978-0-387-87708-2

[B16] Faber G. M. Rudy Y. (2000). Action potential and contractility changes in [Na^+^]*_i_* overloaded cardiac myocytes: a simulation study. Biophys. J. 78 (5), 2392–2404. 10.1016/S0006-3495(00)76783-X 10777735 PMC1300828

[B17] Fine B. Vunjak-Novakovic G. (2017). Shortcomings of animal models and the rise of engineered human cardiac tissue. ACS Biomaterials Sci. Eng. 3 (9), 1884–1897. 10.1021/acsbiomaterials.6b00662 33440547

[B18] Franzone P. C. Pavarino L. F. Scacchi S. (2014). Mathematical cardiac electrophysiology Vol. 13 (Cham, Switzerland: Springer). 10.1007/978-3-319-04801-7

[B19] Friel D. D. (1995). [Ca^2+^]*_i_* oscillations in sympathetic neurons: an experimental test of a theoretical model. Biophys. J. 68 (5), 1752–1766. 10.1016/S0006-3495(95)80352-8 7612818 PMC1282078

[B20] Garg P. Garg V. Shrestha R. Sanguinetti M. C. Kamp T. J. Wu J. C. (2018). Human induced pluripotent stem cell–derived cardiomyocytes as models for cardiac channelopathies. Circ. Res. 123 (2), 224–243. 10.1161/CIRCRESAHA.118.311209 29976690 PMC6136439

[B21] Gong J. Q. X. Sobie E. A. (2018). Population-based mechanistic modeling allows for quantitative predictions of drug responses across cell types. NPJ Syst. Biol. Appl. 4 (1), 11. 10.1038/s41540-018-0047-2 29507757 PMC5825396

[B22] Grandi E. Pasqualini F. S. Bers D. M. (2010). A novel computational model of the human ventricular action potential and Ca transient. J. Mol. Cell. Cardiol. 48 (1), 112–121. 10.1016/j.yjmcc.2009.09.019 19835882 PMC2813400

[B23] Hille B. (2001). Ion channels of excitable membranes Vol. 507 (Sinauer Sunderland, MA: Oxford University Press Inc).

[B24] Hund T. J. Kucera J. P. Otani N. F. Rudy Y. (2001). Ionic charge conservation and long-term steady state in the Luo-Rudy dynamic cell model. Biophys. J. 81 (6), 3324–3331. 10.1016/S0006-3495(01)75965-6 11720995 PMC1301789

[B25] Izhikevich E. M. (2007). Dynamical Systems in Neuroscience (Cambridge, MA: MIT Press). 10.7551/mitpress/2526.001.0001

[B26] Jæger K. H. Wall S. Tveito A. (2019a). Detecting undetectables: Can conductances of action potential models be changed without appreciable change in the transmembrane potential? Chaos: Interdiscip. J. Nonlinear Sci. 29 (7), 073102. 10.1063/1.5087629 31370420

[B27] Jæger K. H. Edwards A. G. McCulloch A. Tveito A. (2019b). Properties of cardiac conduction in a cell-based computational model. PloS Comput. Biol. 15 (5), e1007042. 10.1371/journal.pcbi.1007042 31150383 PMC6561587

[B28] Jiang Y. Park P. Hong S.-M. Ban K. (2018). Maturation of cardiomyocytes derived from human pluripotent stem cells: current strategies and limitations. Molecules Cells 41 (7), 613. 10.14348/molcells.2018.0143 29890820 PMC6078855

[B29] Keller H. B. (1987). Lectures on numerical methods in bifurcation problems. Appl. Math. 217, 50.

[B30] Kernik D. C. Morotti S. Di Wu H. Garg P. Duff H. J. Kurokawa J. (2019). A computational model of induced pluripotent stem-cell derived cardiomyocytes incorporating experimental variability from multiple data sources. J. Physiol. 597 (17), 4533–4594. 10.1113/JP277724 31278749 PMC6767694

[B31] Kramer J. Obejero-Paz C. A. Myatt G. Kuryshev Y. A. Bruening-Wright A. Verducci J. S. (2013). MICE models: superior to the HERG model in predicting Torsade de Pointes. Sci. Rep. 3, 2100. 10.1038/srep02100 23812503 PMC3696896

[B32] Liang P. Lan F. Lee A. S. Gong T. Sanchez-Freire V. Wang Y. (2013). Drug screening using a library of human induced pluripotent stem cell-derived cardiomyocytes reveals disease specific patterns of cardiotoxicity. Circulation 127 (16), 1677–1691. 10.1161/CIRCULATIONAHA.113.001883 23519760 PMC3870148

[B33] Liesen J. Mehrmann V. (2015). Linear Algebra (Cham, Switzerland: Springer). 10.1007/978-3-319-24346-7

[B34] Luo C.-H. Rudy Y. (1994). A dynamic model of the cardiac ventricular action potential. i. simulations of ionic currents and concentration changes. Circ. Res. 74 (6), 1071–1096. 10.1161/01.RES.74.6.1071 7514509

[B35] Lyche T. (2017). Numerical Linear Algebra and Matrix Factorizations (Oslo, Norway: University of Oslo, lecture notes).

[B36] Ma J. Guo L. Fiene S. J. Anson B. D. Thomson J. A. Kamp T. J. (2011). High purity human-induced pluripotent stem cell-derived cardiomyocytes: electrophysiological properties of action potentials and ionic currents. Am. J. Physiology-Heart Circulatory Physiol. 301 (5), H2006–H2017. 10.1152/ajpheart.00694.2011 PMC4116414 21890694

[B37] Mathur A. Loskill P. Shao K. Huebsch N. Hong S. Marcus S. G. (2015). Human iPSC-based cardiac microphysiological system for drug screening applications. Sci. Rep. 5, 8883. 10.1038/srep08883 25748532 PMC4352848

[B38] Mathur A. Ma Z. Loskill P. Jeeawoody S. Healy K. E. (2016). In vitro cardiac tissue models: current status and future prospects. Adva. Drug Deliv Rev. 96, 203–213. 10.1016/j.addr.2015.09.011 PMC4832980 26428618

[B39] Mirams G. R. Cui Y. Sher A. Fink M. Cooper J. Heath B. M. (2011). Simulation of multiple ion channel block provides improved early prediction of compounds’ clinical torsadogenic risk. Cardiovasc. Res. 91 (1), 53–61. 10.1093/cvr/cvr044 21300721 PMC3112019

[B40] Mohammad S. Zhou Z. Gong Q. January C. T. (1997). Blockage of the HERG human cardiac K^+^ channel by the gastrointestinal prokinetic agent cisapride. Am. J. Physiology-Heart Circulatory Physiol. 273 (5), H2534–H2538. 10.1152/ajpheart.1997.273.5.H2534 9374794

[B41] Moody W. J. Bosma M. M. (2005). Ion channel development, spontaneous activity, and activity-dependent development in nerve and muscle cells. Physiol. Rev. 85 (3), 883–941. 10.1152/physrev.00017.2004 15987798

[B42] Mora C. Serzanti M. Consiglio A. Memo M. Dell’Era P. (2017). Clinical potentials of human pluripotent stem cells. Cell Biol. Toxicol. 33 (4), 351–360. 10.1007/s10565-017-9384-y 28176010

[B43] Nelder J. A. Mead R. (1965). A simplex method for function minimization. Comput. J. 7 (4), 308–313. 10.1093/comjnl/7.4.308

[B44] O’Hara T. Virág L. Varró A. Rudy Y. (2011). Simulation of the undiseased human cardiac ventricular action potential: Model formulation and experimental validation. PloS Comput. Biol. 7 (5), e1002061. 10.1371/journal.pcbi.1002061 21637795 PMC3102752

[B45] Orchard C. H. Pásek M. Brette F. (2009). The role of mammalian cardiac t-tubules in excitation–contraction coupling: experimental and computational approaches. Exp. Physiol. 94 (5), 509–519. 10.1113/expphysiol.2008.043984 19297389

[B46] Paci M. Hyttinen J. Aalto-Setälä K. Severi S. (2013). Computational models of ventricular-and atrial-like human induced pluripotent stem cell derived cardiomyocytes. Ann. Biomed. Eng. 41 (11), 2334–2348. 10.1007/s10439-013-0833-3 23722932

[B47] Paci M. Hyttinen J. Rodriguez B. Severi S. (2015). Human induced pluripotent stem cell-derived versus adult cardiomyocytes: an *in silico* electrophysiological study on effects of ionic current block. Br. J. Pharmacol. 172 (21), 5147–5160. 10.1111/bph.13282 26276951 PMC4629192

[B48] Paci M. Passini E. Severi S. Hyttinen J. Rodriguez B. (2017). Phenotypic variability in LQT3 human induced pluripotent stem cell-derived cardiomyocytes and their response to anti-arrhythmic pharmacological therapy: an in silico approach. Heart Rhythm 14 (11), 1704–1712. 10.1016/j.hrthm.2017.07.026 28756098 PMC5668441

[B49] Paci M. Pölönen R.-P. Cori D. Penttinen K. Aalto-Setälä K. Severi S. (2018). Automatic optimization of an *in silico* model of human iPSC derived cardiomyocytes recapitulating calcium handling abnormalities. Front. In Physiol. 9, 709. 10.3389/fphys.2018.00709 29997516 PMC6028769

[B50] Plonsey R. Barr R. C. (2007). Bioelectricity, A Quantitative Approach (New York, USA: Springer).

[B51] Poulet C. Wettwer E. Grunnet M. Jespersen T. Fabritz L. Matschke K. (2015). Late sodium current in human atrial cardiomyocytes from patients in sinus rhythm and atrial fibrillation. PloS One 10 (6), e0131432–e0131432. 10.1371/journal.pone.0131432 26121051 PMC4485891

[B52] Qu Z. Hu G. Garfinkel A. Weiss J. N. (2014). Nonlinear and stochastic dynamics in the heart. Phys. Rep. 543 (2), 61–162. 10.1016/j.physrep.2014.05.002 25267872 PMC4175480

[B53] Quarteroni A. Lassila T. Rossi S. Ruiz-Baier R. (2017). Integrated heart—coupling multiscale and multiphysics models for the simulation of the cardiac function. Comput. Methods In Appl. Mechanics Eng. 314, 345–407. 10.1016/j.cma.2016.05.031

[B54] Rice J. J. Jafri M. S. Winslow R. L. (1999). Modeling gain and gradedness of Ca^2+^ release in the functional unit of the cardiac diadic space. Biophys. J. 77 (4), 1871–1884. 10.1016/S0006-3495(99)77030-X 10512809 PMC1300470

[B55] Rodriguez B. Carusi A. Abi-Gerges N. Ariga R. Britton O. Bub G. (2015). Human-based approaches to pharmacology and cardiology: an interdisciplinary and intersectorial workshop. Europace 18 (9), 1287–1298. 10.1093/europace/euv320 26622055 PMC5006958

[B56] Ronaldson-Bouchard K. Ma S. P. Yeager K. Chen T. Song L. Sirabella D. (2018). Advanced maturation of human cardiac tissue grown from pluripotent stem cells. Nature 556 (7700), 239. 10.1038/s41586-018-0016-3 29618819 PMC5895513

[B57] Rudy Y. Silva J. R. (2006). Computational biology in the study of cardiac ion channels and cell electrophysiology. Q. Rev. Biophys. 39 (01), 57–116. 10.1017/S0033583506004227 16848931 PMC1994938

[B58] Rudy Y. (2012). From genes and molecules to organs and organisms: Heart. Compr. Biophys. 9, 268–327. 10.1016/B978-0-12-374920-8.00924-3

[B59] Sala L. Bellin M. Mummery C. L. (2017). Integrating cardiomyocytes from human pluripotent stem cells in safety pharmacology: has the time come? Br. J. Pharmacol. 174 (21), 3749–3765. 10.1111/bph.13577 27641943 PMC5647193

[B60] Sobie E. A. Jafri M. S. Lederer W. J. (2010). “Models of cardiac Ca^2+^-induced Ca^2+^ release and Ca^2+^ sparks,” in Understanding Calcium Dynamics. Experiments and Theory., chapter 6. Eds. Falcke M. Malchow D. (Berlin Heidelberg, Germany: Springer-Verlag, Berlin Heidelberg), 97–118. 10.1007/978-3-540-44878-5_6

[B61] Sontheimer H. Ransom B. R. Waxman S. G. (1992). Different Na^+^ currents in P0-and P7-derived hippocampal astrocytes *in vitro*: evidence for a switch in Na^+^ channel expression *in vivo*. Brain Res. 597 (1), 24–29. 10.1016/0006-8993(92)91501-5 1335819

[B62] Sterratt D. Graham B. Gillies A. Willshaw D. (2011). Principles of Computational Modelling in Neuroscience (New York, USA: Cambridge University Press).

[B63] Ten Tusscher K. H. W. J. Panfilov A. V. (2006). Cell model for efficient simulation of wave propagation in human ventricular tissue under normal and pathological conditions. Phys. In Med. Biol. 51 (23), 6141. 10.1088/0031-9155/51/23/014 17110776

[B64] Tveito A. Lines G. T. (2016). Computing Characterizations of Drugs for Ion Channels and Receptors Using Markov Models Vol. 111 (Cham, Switzerland: Springer-Verlag, Lecture Notes). 10.1007/978-3-319-30030-6

[B65] Tveito A. Lines G. T. Li P. McCulloch A. (2011). Defining candidate drug characteristics for long-QT (LQT3) syndrome. Math. Biosci. Eng. 8 (3), 861–873. 10.3934/mbe.2011.8.861 21675815

[B66] Tveito A. Jæger K. H. Kuchta M. Mardal K.-A. Rognes M. E. (2017). A cell-based framework for numerical modeling of electrical conduction in cardiac tissue. Front. In Phys. 5, 48. 10.3389/fphy.2017.00048

[B67] Tveito A. Jæger K. H. Huebsch N. Charrez B. Edwards A. G. Wall S. (2018). Inversion and computational maturation of drug response using human stem cell derived cardiomyocytes in microphysiological systems. Sci. Rep. 8 (1), 17626. 10.1038/s41598-018-35858-7 30514966 PMC6279833

[B68] Tveito A. Maleckar M. M. Lines G. T. (2018). Computing optimal properties of drugs using mathematical models of single channel dynamics. Comput. Math. Biophys. 6 (1), 41–64. 10.1515/cmb-2018-0004

[B69] Wilders R. (2007). Computer modelling of the sinoatrial node. Med. Biol. Eng. Comput. 45 (2), 189–207. 10.1007/s11517-006-0127-0 17115219

[B70] Ye L. Ni X. Zhao Z.-A. Lei W. Hu S. (2018). The application of induced pluripotent stem cells in cardiac disease modeling and drug testing. J. Cardiovasc. Trans. Res. 11 (5), 366–374. 10.1007/s12265-018-9811-3 29845439

[B71] Yoshida Y. Yamanaka S. (2017). Induced pluripotent stem cells 10 years later. Circ. Res. 120 (12), 1958–1968. 10.1161/CIRCRESAHA.117.311080 28596174

[B72] Zemzemi N. Bernabeu M. O. Saiz J. Cooper J. Pathmanathan P. Mirams G. R. (2013). Computational assessment of drug-induced effects on the electrocardiogram: from ion channel to body surface potentials. Br. J. Pharmacol. 168 (3), 718–733. 10.1111/j.1476-5381.2012.02200.x 22946617 PMC3579290

[B73] Zhabyeyev P. Missan S. Jones S. E. McDonald T. F. (2000). Low-affinity block of cardiac K^+^ currents by nifedipine. Eur. J. Pharmacol. 401 (2), 137–143. 10.1016/S0014-2999(00)00413-1 10924918

[B74] Zhang S. Zhou Z. Gong Q. Makielski J. C. January C. T. (1999). Mechanism of block and identification of the verapamil binding domain to HERG potassium channels. Circ. Res. 84 (9), 989–998. 10.1161/01.RES.84.9.989 10325236

[B75] Zhao Z. Lan H. El-Battrawy I. Li X. Buljubasic F. Sattler K. (2018). et al. Ion channel expression and characterization in human induced pluripotent stem cell-derived cardiomyocytes. Stem Cells Int. 2018, 6067096. 10.1155/2018/6067096 29535773 PMC5835237

