# Supplementary material for: Improved Computational Identification of Drug Response Using Optical Measurements of Human Stem Cell Derived Cardiomyocytes in Microphysiological Systems
Source: Front Pharmacol. 2020 Feb 12;10:1648. doi: 10.3389/fphar.2019.01648 (PMC7029356; doi:10.3389/fphar.2019.01648)
Supplement: Supplementary file 1 [file DataSheet_1.pdf]

Supplementary information

Contents

S1 Summary of the base model formulation **3**
S1.1 The membrane potential 3
S1.2 Membrane currents 3
S1.3 Ca^{2+} dynamics 7
S1.4 Ca^{2+} fluxes 7
S1.5 Nernst equilibrium potentials 9

S2 Details of the Ca^{2+} dynamics model **14**
S2.1 Description of the Ca^{2+} fluxes of the base model 14
S2.1.1 Flux through the SERCA pumps (J^n_c) 16
S2.1.2 Passive diffusion fluxes between compartments (J^c_d, J^c_{sl}, and J^s_n) 17
S2.1.3 Buffer fluxes (J^b_d, J^b_{sl}, J^b_c, and J^b_s) 18
S2.1.4 Membrane fluxes (J_{CaL}, J_{bCa}, J_{pCa}, and J_{NaCa}) 19
S2.2 Notes on modeling Ca^{2+} dynamics 21
S2.2.1 Ca^{2+}-induced Ca^{2+} release (CICR) 22
S2.2.2 High gain and graded release 22
S2.2.3 Restoring the Ca^{2+} concentration 22
S2.2.4 Common pool models 23
S2.2.5 Local control models 24
S2.2.6 CICR in the base model 24

S3 Details of the inversion procedure **25**
S3.1 Definition of the cost function 25
S3.1.1 Action potential and Ca^{2+} transient durations 25
S3.1.2 Integral of the membrane potential 26
S3.1.3 Norm of the Ca^{2+} transient difference 27
S3.1.4 Upstroke velocity 27
S3.1.5 Ca^{2+} transient amplitude 27

S3.1.6 Maximum and resting values of the membrane potential and Ca^{2+} concentration 28
S3.1.7 Information about individual currents 29
S3.1.8 Ca^{2+} balance 29
S3.1.9 Regularization of adjustment factors 29
S3.2 Specification of the cost function weights 30

S4 Supplementary tables 31

S5 Supplementary figures 32

S1 Summary of the base model formulation

In this section, we describe the full base model formulation. In this formulation, the membrane potential (v) is given in units of mV, and the Ca^{2+} concentrations are given in units of mM. All currents are expressed in units of A/F, and the Ca^{2+} fluxes are expressed as mmol/ms per total cell volume (i.e., in units of mM/ms). Time is given in ms. The parameters of the model are given in Tables S1–S6.

S1.1 The membrane potential

The membrane potential is governed by the equation

$$\frac{dv}{dt} = -(I_{\mathrm{Na}} + I_{\mathrm{NaL}} + I_{\mathrm{CaL}} + I_{\mathrm{to}} + I_{\mathrm{Kr}} + I_{\mathrm{Ks}} + I_{\mathrm{K1}} + I_{\mathrm{NaCa}} + I_{\mathrm{NaK}} + I_{\mathrm{pCa}} + I_{\mathrm{bCl}} + I_{\mathrm{bCa}} + I_{\mathrm{f}} + I_{\mathrm{stim}}), \quad (1)$$

where I_{stim} is an applied stimulus current, and I_{Na}, I_{NaL}, I_{CaL}, I_{to}, I_{Kr}, I_{Ks}, I_{K1}, I_{NaCa}, I_{NaK}, I_{pCa}, I_{bCl}, I_{bCa}, and I_{f} are membrane currents specified below. In our simulations, I_{stim} is given as a constant current of size -40 A/F for adult cells and -5 A/F for hiPSC-CMs. The I_{stim} current is applied until the membrane potential reaches a value of -40 mV.

S1.2 Membrane currents

The currents through the voltage-gated ion channels on the cell membrane are in general given on the form

$$I = go(v - E),$$

where g is the channel conductance, v is the membrane potential and E is the equilibrium potential of the channel. Furthermore, $o = \prod_i z_i$ is the open probability of the channels, where z_i are gating variables, either given as a function of the membrane potential or governed by equations of the form

$$z_i' = \frac{1}{\tau_{z_i}}(z_{i,\infty} - z_i). \quad (2)$$

The parameters τ_{z_i} and $z_{i,\infty}$ are specified for each of the gating variables of the model in Table S7.

Fast sodium current The formulation of the fast sodium current is an adjusted version of the model given in [1], supporting slower upstroke velocities more similar to those observed in the optical measurements of hiPSC-CMs. The current is given by

$$I_{\mathrm{Na}} = g_{\mathrm{Na}} o_{\mathrm{Na}} (v - E_{\mathrm{Na}}), \tag{3}$$

where the open probability is given by

$$o_{\mathrm{Na}} = m^3 j, \tag{4}$$

and m and j are gating variables governed by equations of the form (2).

Late sodium current The formulation of the late sodium current, I_{NaL}, is based on [2] and is given by

$$I_{\mathrm{NaL}} = g_{\mathrm{NaL}} o_{\mathrm{NaL}} (v - E_{\mathrm{Na}}), \tag{5}$$

where the open probability is given by

$$o_{\mathrm{NaL}} = m_L h_L, \tag{6}$$

and m_L and h_L are gating variables governed by equations of the form (2).

Transient outward potassium current The formulation of the transient outward potassium current, I_{to}, is based on [3] and is given by

$$I_{\mathrm{to}} = g_{\mathrm{to}} o_{\mathrm{to}} (v - E_{\mathrm{to}}), \tag{7}$$

where the open probability is given by

$$o_{\mathrm{to}} = q_{\mathrm{to}} r_{\mathrm{to}}, \tag{8}$$

and q_{to} and r_{to} are gating variables governed by equations of the form (2).

Rapidly activating potassium current The formulation of the rapidly activating potassium current, I_{Kr}, is based on [3] and is given by

$$I_{\mathrm{Kr}} = g_{\mathrm{Kr}} o_{\mathrm{Kr}} (v - E_{\mathrm{K}}), \tag{9}$$

where

$$o_{\mathrm{Kr}} = x_{\mathrm{Kr1}} x_{\mathrm{Kr2}}, \tag{10}$$

and the dynamics of x_{Kr1} and x_{Kr2} are governed by equations of the form (2).

Slowly activating potassium current The formulation of the slowly activating potassium current, I_{Ks}, is based on [1] and is given by

$$I_{\mathrm{Ks}} = g_{\mathrm{Ks}} o_{\mathrm{Ks}} (v - E_{\mathrm{Ks}}), \tag{11}$$

where

$$o_{\mathrm{Ks}} = x_{\mathrm{Ks}}^2, \tag{12}$$

and the dynamics of x_{Ks} is governed by an equation of the form (2).

Inward rectifier potassium current The formulation of the inward rectifier potassium current, I_{K1}, is based on [1] and is given by

$$I_{\mathrm{K1}} = g_{\mathrm{K1}} o_{\mathrm{K1}} (v - E_{\mathrm{K}}), \tag{13}$$

where

$$o_{\mathrm{K1}} = \frac{a_{\mathrm{K1}}}{a_{\mathrm{K1}} + b_{\mathrm{K1}}}, \tag{14}$$

$$a_{\mathrm{K1}} = \frac{3.9}{1 + e^{0.6(v - E_K - 200)}}, \tag{15}$$

$$b_{\mathrm{K1}} = \frac{-1.5e^{0.0002(v - E_K + 100)} + e^{0.6(v - E_K - 10)}}{1 + e^{0.45(v - E_K)}}. \tag{16}$$

Hyperpolarization activated funny current The formulation for the hyperpolarization activated funny current, I_{f}, is based on [3] and is given by

$$I_{\mathrm{f}} = g_{\mathrm{f}} o_{\mathrm{f}} (v - E_{\mathrm{f}}), \tag{17}$$

where

$$o_{\mathrm{f}} = x_{\mathrm{f}}, \tag{18}$$

and the dynamics of x_{f} is governed by an equation of the form (2).

L-type $\mathbf{Ca^{2+}}$ current The formualtion for the L-type Ca^{2+} current, I_{CaL}, is based on the formulation in [1] and is given by

$$I_{\mathrm{CaL}} = g_{\mathrm{CaL}} o_{\mathrm{CaL}} \frac{(2F)^2 v}{RT} \frac{0.341 c_d e^{\frac{2Fv}{RT}} - 0.341 c_e}{e^{\frac{2Fv}{RT}} - 1}, \tag{19}$$

where

$$o_{\mathrm{CaL}} = df(1 - f_{\mathrm{Ca}}), \tag{20}$$

and the dynamics of d, f and f_{Ca} are governed by equations of the form (2).

Background currents The formulation of the background currents, I_{bCa} and I_{bCl}, are based on [1] and are given by

$$I_{\text{bCa}} = g_{\text{bCa}}(v - E_{\text{Ca}}), \tag{21}$$

$$I_{\text{bCl}} = g_{\text{bCl}}(v - E_{\text{Cl}}). \tag{22}$$

Sodium-calcium exchanger The formulation of the Na^+-Ca^{2+} exchanger current, I_{NaCa}, is based on [1] and is given by

$$I_{\text{NaCa}} = \bar{I}_{\text{NaCa}} \frac{e^{\frac{\nu F v}{RT}} [\text{Na}^+]_i^3 c_e - e^{\frac{(\nu-1)Fv}{RT}} [\text{Na}^+]_e^3 c_{sl}}{s_{\text{NaCa}} \left(1 + \left(\frac{K_{\text{act}}}{c_{sl}}\right)^2\right) \left(1 + k_{\text{sat}} e^{\frac{(\nu-1)Fv}{RT}}\right)}, \tag{23}$$

where

$$s_{\text{NaCa}} = K_{\text{Ca},i} [\text{Na}^+]_e^3 \left(1 + \left(\frac{[\text{Na}^+]_i}{K_{\text{Na},i}}\right)^3\right) + K_{\text{Na},e}^3 c_{sl} \left(1 + \frac{c_{sl}}{K_{\text{Ca},i}}\right) + K_{\text{Ca},e} [\text{Na}^+]_i^3 + [\text{Na}^+]_i^3 c_e + [\text{Na}^+]_e^3 c_{sl}.$$

Sarcolemmal Ca^{2+} pump The formulation of the current through the sarcolemmal Ca^{2+} pump, I_{pCa}, is based on [1] and is given by

$$I_{\text{pCa}} = \bar{I}_{\text{pCa}} \frac{c_{sl}^2}{K_{\text{pCa}}^2 + c_{sl}^2}. \tag{24}$$

Sodium-potassium pump The current through the Na^+-K^+ pump, I_{NaK}, is based on [1] and is given by

$$I_{\text{NaK}} = \bar{I}_{\text{NaK}} \frac{f_{\text{NaK}}}{1 + \left(\frac{K_{\text{Na,i}}^{\text{NaK}}}{[\text{Na}^+]_{\text{i}}}\right)^4} \frac{[\text{K}^+]_{\text{e}}}{[\text{K}^+]_{\text{e}} + K_{\text{K,e}}}, \tag{25}$$

where

$$f_{\text{NaK}} = \frac{1}{1 + 0.12 e^{-0.1 \frac{Fv}{RT}}} + \frac{0.037}{7} \left(e^{\frac{[\text{Na}^+]_{\text{e}}}{67}} - 1\right) e^{-\frac{Fv}{RT}}. \tag{26}$$

S1.3 Ca^{2+} dynamics

The Ca^{2+} dynamics are governed by

$$\frac{dc_d}{dt} = \frac{1}{V_d}(J_{\text{CaL}} - J_d^b - J_d^c), \qquad \frac{db_d}{dt} = \frac{1}{V_d}J_d^b, \tag{27}$$

$$\frac{dc_{sl}}{dt} = \frac{1}{V_{sl}}(J_e^{sl} - J_{sl}^c - J_{sl}^b + J_s^{sl}), \qquad \frac{db_{sl}}{dt} = \frac{1}{V_{sl}}J_{sl}^b, \tag{28}$$

$$\frac{dc_c}{dt} = \frac{1}{V_c}(J_{sl}^c + J_d^c - J_c^n - J_c^b), \qquad \frac{db_c}{dt} = \frac{1}{V_c}J_c^b, \tag{29}$$

$$\frac{dc_s}{dt} = \frac{1}{V_s}(J_n^s - J_s^{sl} - J_s^b), \qquad \frac{db_s}{dt} = \frac{1}{V_s}J_s^b, \tag{30}$$

$$\frac{dc_n}{dt} = \frac{1}{V_n}(J_c^n - J_n^s), \tag{31}$$

where c_d is the concentration of free Ca^{2+} in the dyad, b_d is the concentration of Ca^{2+} bound to a buffer in the dyad, c_{sl} is the concentration of free Ca^{2+} in the SL compartment, b_{sl} is the concentration of Ca^{2+} bound to a buffer in the SL compartment, c_c is the concentration of free Ca^{2+} in the bulk cytosol, b_c is the concentration of Ca^{2+} bound to a buffer in the bulk cytosol, c_s is the concentration of free Ca^{2+} in the jSR, b_s is the concentration of Ca^{2+} bound to a buffer in the jSR, and c_n is the concentration of free Ca^{2+} in the nSR. The expressions for the fluxes are specified below.

S1.4 Ca^{2+} fluxes

Flux through the SERCA pumps The flux from the bulk cytosol to the nSR through the SERCA pumps is given by

$$J_c^n = \bar{J}_{\text{SERCA}} \frac{\left(\frac{c_c}{K_c}\right)^2 - \left(\frac{c_n}{K_n}\right)^2}{1 + \left(\frac{c_c}{K_c}\right)^2 + \left(\frac{c_n}{K_n}\right)^2}. \tag{32}$$

Flux through the RyRs The flux from the jSR to the SL compartment is given by

$$J_s^{sl} = J_{\text{RyR}} + J_{\text{leak}}, \tag{33}$$

where J_{RyR} represents the flux through the active RyR channels and J_{leak} represents the flux through the RyR channels that are always open, given by

$$J_{\text{RyR}} = p \cdot r \cdot \alpha_{\text{RyR}}(c_s - c_{sl}), \tag{34}$$

$$J_{\text{leak}} = \gamma_{\text{RyR}} \cdot \alpha_{\text{RyR}}(c_s - c_{sl}), \tag{35}$$

respectively. Here, p is the open probability of the active RyR channels given by

$$p = \frac{c_d^3}{c_d^3 + \kappa_{\mathrm{RyR}}^3}, \tag{36}$$

and r represents the fraction of RyR channels that are not inactivated and is governed by the equation

$$\frac{dr}{dt} = -\frac{J_{\mathrm{RyR}}}{\beta_{\mathrm{RyR}}} + \frac{\eta_{\mathrm{RyR}}}{p}(1-r). \tag{37}$$

Passive diffusion fluxes between compartments The passive diffusion fluxes between compartments are given by

$$J_d^c = \alpha_d^c(c_d - c_c), \tag{38}$$
$$J_{sl}^c = \alpha_{sl}^c(c_{sl} - c_c), \tag{39}$$
$$J_n^s = \alpha_n^s(c_n - c_s). \tag{40}$$

Buffer fluxes The fluxes of free Ca^{2+} binding to a Ca^{2+} buffer are given by

$$J_d^b = V_d(k_{\mathrm{on}}^d c_d(B_{\mathrm{tot}}^d - b_d) - k_{\mathrm{off}}^d b_d), \tag{41}$$
$$J_{sl}^b = V_{sl}(k_{\mathrm{on}}^{sl} c_{sl}(B_{\mathrm{tot}}^{sl} - b_{sl}) - k_{\mathrm{off}}^{sl} b_{sl}), \tag{42}$$
$$J_c^b = V_c(k_{\mathrm{on}}^c c_c(B_{\mathrm{tot}}^c - b_c) - k_{\mathrm{off}}^c b_c), \tag{43}$$
$$J_s^b = V_s(k_{\mathrm{on}}^s c_s(B_{\mathrm{tot}}^s - b_s) - k_{\mathrm{off}}^s b_s). \tag{44}$$

Membrane fluxes The membrane fluxes, J_{CaL}, J_{bCa}, J_{pCa}, and J_{NaCa}, are given by

$$J_{\mathrm{CaL}} = -\frac{\chi C_m}{2F} I_{\mathrm{CaL}}, \qquad J_{\mathrm{pCa}} = -\frac{\chi C_m}{2F} I_{\mathrm{pCa}}, \tag{45}$$
$$J_{\mathrm{bCa}} = -\frac{\chi C_m}{2F} I_{\mathrm{bCa}}, \qquad J_{\mathrm{NaCa}} = \frac{\chi C_m}{F} I_{\mathrm{NaCa}}, \tag{46}$$

where I_{CaL}, I_{bCa}, I_{pCa}, and I_{NaCa} are defined by the expressions given above. Furthermore,

$$J_e^{sl} = J_{\mathrm{NaCa}} + J_{\mathrm{pCa}} + J_{\mathrm{bCa}}. \tag{47}$$

Parameter	Description	Value
V_d	Volume fraction of the dyadic subspace	0.001
V_{sl}	Volume fraction of the SL compartment	0.028
V_c	Volume fraction of the bulk cytosol	0.917
V_s	Volume fraction of the jSR	0.004
V_n	Volume fraction of the nSR	0.05
χ	Cell surface to volume ratio	0.6 μm^{-1}

Table S1: Default geometry parameters of the base model.

S1.5 Nernst equilibrium potentials

The Nernst equilibrium potentials for the ion channels are defined as

$$E_{\mathrm{Na}} = \frac{RT}{F}\log\left(\frac{[\mathrm{Na}^+]_\mathrm{e}}{[\mathrm{Na}^+]_\mathrm{i}}\right), \tag{48}$$

$$E_{\mathrm{Ca}} = \frac{RT}{2F}\log\left(\frac{[\mathrm{Ca}^{2+}]_\mathrm{e}}{c_{sl}}\right), \tag{49}$$

$$E_{\mathrm{K}} = \frac{RT}{F}\log\left(\frac{[\mathrm{K}^+]_\mathrm{e}}{[\mathrm{K}^+]_\mathrm{i}}\right), \tag{50}$$

$$E_{\mathrm{Ks}} = \frac{RT}{F}\log\left(\frac{[\mathrm{K}^+]_\mathrm{e} + 0.018[\mathrm{Na}^+]_\mathrm{e}}{[\mathrm{K}^+]_\mathrm{i} + 0.018[\mathrm{Na}^+]_\mathrm{i}}\right), \tag{51}$$

$$E_{\mathrm{Cl}} = \frac{RT}{F}\log\left(\frac{[\mathrm{Cl}^+]_\mathrm{e}}{[\mathrm{Cl}^+]_\mathrm{i}}\right), \tag{52}$$

$$E_{\mathrm{f}} = -17\ \mathrm{mV}, \tag{53}$$

for the parameter values given in Table S2.

Parameter	Description	Value
C_m	Specific membrane capacitance	0.01 μF/μm^2
F	Faraday's constant	96.485 C/mmol
R	Universal gas constant	8.314 J/(mol·K)
T	Temperature	310 K
$[Ca^{2+}]_e$	Extracellular Ca^{2+} concentration	1.8 mM
$[Na^+]_e$	Extracellular sodium concentration	140 mM
$[Na^+]_i$	Intracellular sodium concentration	8 mM
$[K^+]_e$	Extracellular potassium concentration	5.4 mM
$[K^+]_i$	Intracellular potassium concentration	120 mM
$[Cl^-]_e$	Extracellular chloride concentration	150 mM
$[Cl^-]_i$	Intracellular chloride concentration	15 mM

Table S2: Physical constants and ionic concentrations of the base model.

Parameter	Value	Parameter	Value
g_{Na}	12.6 mS/μF	g_{CaL}	0.12 nL/(μF ms)
g_{NaL}	0.025 mS/μF	g_{bCa}	0.00055 mS/μF
g_{to}	0.27 mS/μF	$\bar{I}_{\mathrm{NaCa}}$	4.9 μA/μF
g_{Kr}	0.025 mS/μF	$\bar{I}_{\mathrm{pCa}}$	0.068 μA/μF
g_{Ks}	0.003 mS/μF	$\bar{J}_{\mathrm{SERCA}}$	0.00024 mM/ms
g_{K1}	0.37 mS/μF	α_{RyR}	0.0075 ms^{-1}
g_{f}	0.0001 mS/μF	α_d^c	0.0017 ms^{-1}
g_{bCl}	0.007 mS/μF	α_{sl}^c	0.15 ms^{-1}
$\bar{I}_{\mathrm{NaK}}$	1.8 μA/μF	α_n^s	0.012 ms^{-1}

Table S3: Conductance values and similar parameters for each of the membrane currents and intracellular Ca^{2+} fluxes of the base model.

Parameter	Flux	Value
K_c	J_c^n	0.00025 mM
K_n	J_c^n	1.7 mM
β_{RyR}	J_s^{sl}	0.038 mM
γ_{RyR}	J_s^{sl}	0.001
κ_{RyR}	J_{RyR}	0.015 mM
η_{RyR}	J_s^{sl}	0.00001 ms^{-1}

Table S4: Parameters for the intracellular Ca^{2+} fluxes of the base model.

Parameter	Current	Value
k_{sat}	I_{NaCa}	0.3
ν	I_{NaCa}	0.3
K_{act}	I_{NaCa}	0.00015 mM
$K_{\mathrm{Ca},i}$	I_{NaCa}	0.0036 mM
$K_{\mathrm{Ca},e}$	I_{NaCa}	1.3 mM
$K_{\mathrm{Na},i}$	I_{NaCa}	12.3 mM
$K_{\mathrm{Na},e}$	I_{NaCa}	87.5 mM
$K_{\mathrm{Na},i}^{\mathrm{NaK}}$	I_{NaK}	11 mM
$K_{\mathrm{K},e}$	I_{NaK}	1.5 mM
K_{pCa}	I_{pCa}	0.0005 mM

Table S5: Parameters for the membrane currents of the base model.

Parameter	Compartment	Value
B^c_{tot}	Bulk cytosol	0.07 mM
k^c_{on}	Bulk cytosol	40 $\text{ms}^{-1}\text{mM}^{-1}$
k^c_{off}	Bulk cytosol	0.03 ms^{-1}
B^d_{tot}	Dyad	1.2 mM
k^d_{on}	Dyad	100 $\text{ms}^{-1}\text{mM}^{-1}$
k^d_{off}	Dyad	1 ms^{-1}
B^{sl}_{tot}	Subsarcolemmal space	0.9 mM
k^{sl}_{on}	Subsarcolemmal space	100 $\text{ms}^{-1}\text{mM}^{-1}$
k^{sl}_{off}	Subsarcolemmal space	0.15 ms^{-1}
B^s_{tot}	Junctional SR	27 mM
k^s_{on}	Junctional SR	100 $\text{ms}^{-1}\text{mM}^{-1}$
k^s_{off}	Junctional SR	65 ms^{-1}

Table S6: Parameters for the Ca^{2+} buffers of the base model.

Current	Gate	z_∞	α_z	β_z	τ_z
I_{Na}	m	$\frac{1}{\left(1+e^{(-57-v)/9}\right)^2}$	$0.13e^{-((v+46)/16)^2}$	$0.06e^{-((v-5)/51)^2}$	$\alpha_m+\beta_m$
	j	$\frac{1}{\left(1+e^{(v+72)/7}\right)^2}$	0, if $v \geq -40$<br>$\frac{\left(\begin{array}{c}-2.5\cdot 10^4 e^{0.2v}\\ -7\cdot 10^{-6}e^{-0.04v}\end{array}\right)(v+38)}{1+e^{0.3(v+79)}}$, otherwise	$\frac{0.6e^{0.06v}}{1+e^{-0.1(v+32)}}$, if $v \geq -40$<br>$\frac{0.02e^{-0.01v}}{1+e^{-0.14(v+40)}}$, otherwise	$\frac{1}{\alpha_j+\beta_j}$
I_{NaL}	m_L	$\frac{1}{1+e^{(-43-v)/5}}$	$\frac{1}{6.8e^{(v+12)/35}}$	$8.6e^{-(v+77)/6}$	$\alpha_m+\beta_m$
	h_L	$\frac{1}{1+e^{(v+88)/7.5}}$			200 ms
I_{CaL}	d	$\frac{1}{1+e^{-(v+5)/6}}$	$\frac{1-e^{-\frac{v+5}{6}}}{0.035(v+5)}$		α_d
	f	$\frac{1}{1+e^{(v+35)/9}}+\frac{0.6}{1+e^{(50-v)/20}}$	$\frac{1}{0.02e^{-(0.034(v+14.5)^2)}+0.02}$		α_f
	f_{Ca}	$\frac{1.7c_d}{1.7c_d+0.012}$	$\frac{1}{1.7c_d+0.012}$		α_{Ca}
I_{to}	q_{to}	$\frac{1}{1+e^{(v+53)/13}}$	$\frac{39}{0.57e^{-0.08(v+44)}+0.065e^{0.1(v+46)}}$	6	$\alpha_{q_{\mathrm{to}}}+\beta_{q_{\mathrm{to}}}$
	r_{to}	$\frac{1}{1+e^{-(v-22.3)/18.75}}$	$\frac{14.4}{e^{0.09(v+30.61)}+0.37e^{-0.12(v+24)}}$	2.75	$\alpha_{r_{\mathrm{to}}}+\beta_{r_{\mathrm{to}}}$
I_{Kr}	x_{Kr1}	$\frac{1}{1+e^{-(v+20.7)/4.9}}$	$\frac{450}{1+e^{-(v+45)/10}}$	$\frac{6}{1+e^{(v+30)/11.5}}$	$\alpha_{x_{\mathrm{Kr1}}}\cdot\beta_{x_{\mathrm{Kr1}}}$
	x_{Kr2}	$\frac{1}{1+e^{(v+88)/50}}$	$\frac{3}{1+e^{-(v+60)/20}}$	$\frac{1.12}{1+e^{(v-60)/20}}$	$\alpha_{x_{\mathrm{Kr2}}}\cdot\beta_{x_{\mathrm{Kr2}}}$
I_{Ks}	x_{Ks}	$\frac{1}{1+e^{-(v+3.8)/14}}$	$\frac{990}{1+e^{-(v+2.4)/14}}$		$\alpha_{x_{\mathrm{Ks}}}$
I_{f}	x_{f}	$\frac{1}{1+e^{(v+78)/5}}$	$\frac{1900}{1+e^{(v+15)/10}}$		$\alpha_{x_{\mathrm{Ks}}}$

Table S7: Specification of the parameters z_∞ and τ_z, for $z = m$, j, m_L, h_L, d, f, f_{Ca}, q_{to}, r_{to}, x_{Kr1}, x_{Kr2}, x_{Ks} and x_{f} in the equations for the gating variables (2).

S2 Details of the Ca^{2+} dynamics model

In this section, we describe some details of the model of the intracellular Ca^{2+} dynamics. First, in Section S2.1, we describe the Ca^{2+} fluxes of the base model and how the parameters of these fluxes can be adjusted for different maturity levels. Next, in Section S2.2 we discuss the concepts of high gain and graded release.

S2.1 Description of the Ca^{2+} fluxes of the base model

As noted above, all Ca^{2+} fluxes, J, are defined in terms of the number of ions flowing per time per total cell volume, in units of mM/ms. Accordingly, the size of a flux in mmol/ms is given by $\bar{J} = V_{\text{cell}} J$, where V_{cell} is the cell volume (in L).

Similarly, for a single compartment with volume $\bar{V}_x$ (in L), volume fraction (dimensionless) $V_x = \frac{\bar{V}_x}{V_{\text{cell}}}$ and Ca^{2+} concentration c_x (in mM), the total number of Ca^{2+} ions in the compartment (in mmol) is given by $n_x = c_x \bar{V}_x$. The change in the number of Ca^{2+}-ions in the compartment is given by

$$\frac{dn_x}{dt} = \bar{J}_x, \tag{54}$$

where $\bar{J}_x$ is the flux of ions into the compartment given in mmol/ms. It is also useful to define an associated concentration flux per total cell volume, $J_x = \bar{J}_x / V_{\text{cell}}$ (in mM/ms). Dividing both sides of (54) by the compartment volume $\bar{V}_x$, we obtain the following equation for the change in Ca^{2+} concentration:

$$\frac{dc_x}{dt} = \frac{1}{\bar{V}_x}\bar{J}_x = \frac{1}{\bar{V}_x} V_{\text{cell}} J_x = \frac{1}{V_x} J_x. \tag{55}$$

Expanding this approach to all the compartments and fluxes of the model, we obtain the following system of equations for the intracellular Ca^{2+} dynamics:

$$\begin{aligned}
\frac{dc_d}{dt} &= \frac{1}{V_d}(J_{\text{CaL}} - J_d^b - J_d^c), & \frac{db_d}{dt} &= \frac{1}{V_d} J_d^b, \\
\frac{dc_{sl}}{dt} &= \frac{1}{V_{sl}}(J_e^{sl} - J_{sl}^c - J_{sl}^b + J_s^{sl}), & \frac{db_{sl}}{dt} &= \frac{1}{V_{sl}} J_{sl}^b, \\
\frac{dc_c}{dt} &= \frac{1}{V_c}(J_{sl}^c + J_d^c - J_c^n - J_c^b), & \frac{db_c}{dt} &= \frac{1}{V_c} J_c^b, \\
\frac{dc_s}{dt} &= \frac{1}{V_s}(J_n^s - J_s^{sl} - J_s^b), & \frac{db_s}{dt} &= \frac{1}{V_s} J_s^b, \\
\frac{dc_n}{dt} &= \frac{1}{V_n}(J_c^n - J_n^s).
\end{aligned}$$

Flux	Description
J_s^{sl}	Flux through the RyRs from the jSR to the SL
J_c^n	Flux through the SERCA pumps from the bulk cytosol to the nSR
J_d^c	Passive diffusion flux between the dyad and the bulk cytosol
J_{sl}^c	Passive diffusion flux between the SL and the bulk cytosol
J_n^s	Passive diffusion flux between the nSR and the jSR
J_d^b	Free Ca^{2+} binding to a buffer in the dyad
J_{sl}^b	Free Ca^{2+} binding to a buffer in the SL
J_c^b	Free Ca^{2+} binding to a buffer in the bulk cytosol
J_s^b	Free Ca^{2+} binding to a buffer in the jSR
J_{CaL}	Ca^{2+}-flux through the L-type Ca^{2+} channels from the extracellular space to the dyad
J_{bCa}	Background Ca^{2+} flux from the extracellular space to the SL
J_{pCa}	Ca^{2+} flux through the Ca^{2+} pump between the extracellular space and the SL
J_{NaCa}	Ca^{2+} flux through the Na^+-Ca^{2+} exchanger between the extracellular space and the SL
J_e^{sl}	Total Ca^{2+} flux from the extracellular space to the SL, defined as $J_e^{sl} = J_{\mathrm{bCa}} + J_{\mathrm{pCa}} + J_{\mathrm{NaCa}}$.

Table S8: Ca^{2+} fluxes of the base model. The direction of all membrane fluxes are defined such that a positive flux corresponds to Ca^{2+} ions flowing into the cell.

Each of the fluxes of the base model are summarized in Table S8, and below each of the fluxes are described in more detail (except for J_{RyR}, which is described in the paper)

S2.1.1 Flux through the SERCA pumps (J_c^n)

The flux from the bulk cytosol to the nSR through SERCA is given on the form

$$J_c^n = \frac{N_{\mathrm{SERCA}}}{V_{\mathrm{cell}}} j_{\mathrm{SERCA}}, \tag{56}$$

where N_{SERCA} is the number of SERCA pumps on the membrane of the nSR, V_{cell} is the total cell volume (in L) and j_{SERCA} is the flux through a single SERCA pump (in mmol/ms). The flux through a single pump is given by an expression based on the formulation in the Grandi et al. model [1]:

$$j_{\mathrm{SERCA}} = J_{\mathrm{SERCA}}^{\max,0} \frac{\left(\frac{c_c}{K_c}\right)^2 - \left(\frac{c_n}{K_n}\right)^2}{1 + \left(\frac{c_c}{K_c}\right)^2 + \left(\frac{c_n}{K_n}\right)^2}, \tag{57}$$

where $J_{\mathrm{SERCA}}^{\max,0}$ has unit mmol/ms and K_c and K_n have unit mM. Defining the parameter

$$\bar{J}_{\mathrm{SERCA}} = \frac{N_{\mathrm{SERCA}}}{V_{\mathrm{cell}}} J_{\mathrm{SERCA}}^{\max,0}, \tag{58}$$

with unit mM/ms, the SERCA flux may be written as

$$J_c^n = \bar{J}_{\mathrm{SERCA}} \frac{\left(\frac{c_c}{K_c}\right)^2 - \left(\frac{c_n}{K_n}\right)^2}{1 + \left(\frac{c_c}{K_c}\right)^2 + \left(\frac{c_n}{K_n}\right)^2}. \tag{59}$$

Scaling the SERCA flux Like for the maturation process with respect to sarcolemmal ion channels, we also assume that cells of different levels of maturity may have different geometries and different densities of SERCA pumps, but that the function of each individual SERCA pump is the same.

This means that we assume that the expression for the flux through a single SERCA pump, j_{SERCA}, remains the same, but that the factor $\frac{N_{\mathrm{SERCA}}}{V_{\mathrm{cell}}}$ may differ for different maturity levels. We again represent the change in the SERCA pump density by introducing a scaling factor λ_{SERCA} between one stage of maturity, S_1, to another stage, S_2, such that

$$\frac{N_{\mathrm{SERCA}}^{S_1}}{V_{\mathrm{cell}}^{S_1}} = (1 + \lambda_{\mathrm{SERCA}}) \frac{N_{\mathrm{SERCA}}^{S_2}}{V_{\mathrm{cell}}^{S_2}}, \tag{60}$$

where

$$\frac{N^{S_1}_{\text{SERCA}}}{V^{S_1}_{\text{cell}}}$$

is the SERCA pump density in the model for maturity stage S_1 and

$$\frac{N^{S_2}_{\text{SERCA}}}{V^{S_2}_{\text{cell}}}$$

is the density in the model for maturity stage S_2. In the model formulation, this can be represented on the form

$$J^{n,S_1}_c = (1 + \lambda_{\text{SERCA}})J^{n,S_2}_c, \tag{61}$$

where J^{n,S_1}_c is the expression for the SERCA pump flux in the S_1 state and J^{n,S_2}_c is the expression in the S_2 state.

S2.1.2 Passive diffusion fluxes between compartments (J^c_d, J^c_{sl}, and J^s_n)

Following the approach in e.g. [4], diffusion between compartments is considered to occur, on average, between the center of adjacent compartments. Fick's law of diffusion may then be approximated as

$$J^b_a = \frac{D^b_a A^b_a}{V_{\text{cell}}} \frac{c_a - c_b}{l^b_a}, \tag{62}$$

where D^b_a is the diffusion coefficient (in dm^2/ms) representing the ease with which Ca^{2+} ions flow between the compartments, A^b_a is the area (in dm^2) of the interface between the compartments, c_a and c_b are the Ca^{2+} concentrations of the compartments (in mM), and l^b_a is the distance between the centers of the two compartments (in dm). Again, V_{cell} is the total cell volume (in L), and the flux J^b_a is defined as the number of ions flowing between the compartments per millisecond per total cell volume. Again, in order to reduce the number of parameters, we define the lumped parameter

$$\alpha^b_a = \frac{D^b_a A^b_a}{V_{\text{cell}} l^b_a}, \tag{63}$$

and write the flux as

$$J^b_a = \alpha^b_a (c_a - c_b). \tag{64}$$

We consider passive diffusive fluxes of this form between the dyad and the bulk cytosol, between the SL and the bulk cytosol, and between the nSR

and jSR, and define these fluxes as:

$$J_d^c = \alpha_d^c(c_d - c_c), \tag{65}$$

$$J_{sl}^c = \alpha_{sl}^c(c_{sl} - c_c), \tag{66}$$

$$J_n^s = \alpha_n^s(c_n - c_s). \tag{67}$$

Scaling the diffusive fluxes In the same manner as above, we define adjustment factors λ_a^b for the diffusive fluxes on the form

$$\frac{D_a^{b,S_1} A_a^{b,S_1}}{V_{\text{cell}}^{S_1} l_a^{b,S_1}} = (1 + \lambda_a^b)\frac{D_a^{b,S_2} A_a^{b,S_2}}{V_{\text{cell}}^{S_2} l_a^{b,S_2}}. \tag{68}$$

Here, λ_a^b may represent a change in any of the geometrical properties A_a^b, V_{cell} or l_a^b, a change in the diffusion coefficient D_a^b, or a combination of these changes. This adjustment is represented in the model for each of the diffusive fluxes by

$$J_d^{c,S_1} = (1 + \lambda_d^c) J_d^{c,S_2}, \tag{69}$$

$$J_{sl}^{c,S_1} = (1 + \lambda_{sl}^c) J_{sl}^{c,S_2}, \tag{70}$$

$$J_n^{s,S_1} = (1 + \lambda_n^s) J_n^{s,S_2}, \tag{71}$$

where J_d^{c,S_1}, J_{sl}^{c,S_1}, and J_n^{s,S_1} denote the S_1 fluxes and J_d^{c,S_2}, J_{sl}^{c,S_2}, and J_n^{s,S_2} denote the S_2 fluxes.

S2.1.3 Buffer fluxes (J_d^b, J_{sl}^b, J_c^b, and J_s^b)

The chemical reaction between Ca^{2+} and a buffer may be written as

$$\text{P} + \text{Ca}^{2+} \underset{k_{\text{off}}}{\overset{k_{\text{on}}}{\rightleftarrows}} \text{B}, \tag{72}$$

where P represents the buffering protein and B represents Ca^{2+} bound to the buffer. Here, k_{on} and k_{off} are the rates of the reaction and are given in units of $\text{ms}^{-1}\text{mM}^{-1}$ and ms^{-1}, respectively. If we let B_{tot} denote the total buffer concentration in some compartment, c denote the concentration of free Ca^{2+} and b denote the concentration of Ca^{2+} bound to the buffer, the law of mass action (see e.g., [5]) gives that the rate of decrease in the free Ca^{2+} concentration in the compartment and the rate of increase in the concentration of Ca^{2+} bound to a buffer due to Ca^{2+} -buffer reactions is

$$R = k_{\text{on}} c(B_{\text{tot}} - b) - k_{\text{off}} b, \tag{73}$$

in units of mmol/ms per compartment volume, $\bar{V}_x$. The corresponding flux in terms of mmol/ms per total cell volume, V_{cell}, may be defined as

$$J = \frac{\bar{V}_x}{V_{\text{cell}}} R = V_x(k_{\text{on}} c(B_{\text{tot}} - b) - k_{\text{off}} b). \tag{74}$$

Consequently, the flux of free Ca^{2+} binding to a buffer in the dyad, the SL, the bulk cytosol and the jSR are given by

$$J_d^b = V_d(k_{\text{on}}^d c_d(B_{\text{tot}}^d - b_d) - k_{\text{off}}^d b_d), \tag{75}$$

$$J_{sl}^b = V_{sl}(k_{\text{on}}^{sl} c_{sl}(B_{\text{tot}}^{sl} - b_{sl}) - k_{\text{off}}^{sl} b_{sl}), \tag{76}$$

$$J_c^b = V_c(k_{\text{on}}^c c_c(B_{\text{tot}}^c - b_c) - k_{\text{off}}^c b_c), \tag{77}$$

$$J_s^b = V_s(k_{\text{on}}^s c_s(B_{\text{tot}}^s - b_s) - k_{\text{off}}^s b_s), \tag{78}$$

respectively.

Scaling the Ca^{2+} buffers Like for membrane and SR membrane channels, we assume that cells of different levels of maturity contain the same types of Ca^{2+} buffers, with the same rates k_{on} and k_{off}, but that the concentration of the Ca^{2+} buffers, B_{tot}, may differ for different types of cells. Therefore, we define scaling parameters for the buffer concentrations on the form

$$B_{\text{tot}}^{d,S_1} = (1 + \lambda_B^d) B_{\text{tot}}^{d,S_2}, \tag{79}$$

$$B_{\text{tot}}^{sl,S_1} = (1 + \lambda_B^{sl}) B_{\text{tot}}^{sl,S_2}, \tag{80}$$

$$B_{\text{tot}}^{c,S_1} = (1 + \lambda_B^c) B_{\text{tot}}^{c,S_2}, \tag{81}$$

$$B_{\text{tot}}^{s,S_1} = (1 + \lambda_B^s) B_{\text{tot}}^{s,S_2}, \tag{82}$$

where B_{tot}^{d,S_1}, B_{tot}^{sl,S_1}, B_{tot}^{c,S_1}, and B_{tot}^{s,S_1} are the buffer concentrations in the S_1 model, and B_{tot}^{d,S_2}, B_{tot}^{sl,S_2}, B_{tot}^{c,S_2}, and B_{tot}^{s,S_2} are the buffer concentrations in the S_2 model.

S2.1.4 Membrane fluxes (J_{CaL}, J_{bCa}, J_{pCa}, and J_{NaCa})

The membrane fluxes J_{CaL}, J_{bCa}, J_{pCa}, and J_{NaCa} may be defined from the expressions for the corresponding membrane currents, I_{CaL}, I_{bCa}, I_{pCa}, and I_{NaCa}. Recall from Section 2.2 in the paper that the membrane currents are expressed on the form

$$I_x = \frac{N_x}{AC_m} i_x, \qquad \text{for } x = \text{CaL, bCa, pCa, and NaCa}, \tag{83}$$

where N_x is the total number of channels of type x on the cell membrane, A is the total membrane area (in μm^2), C_m is the specific membrane capacitance (in pF/μm^2) and i_x is the average single-channel current through a channel of type x (in pA). The corresponding membrane fluxes per total cell volume may similarly be defined as

$$J_x = \frac{N_x}{V_{\text{cell}}} j_x, \qquad \text{for } x = \text{CaL, bCa, pCa, and NaCa}, \tag{84}$$

where V_{cell} is the total cell volume (in L), and j_x is the average Ca^{2+} flux through a single channel of type x (in mmol/ms). The average flux of Ca^{2+} through a single Ca^{2+} channel may be written as

$$j_x = -\frac{10^{-15}}{2F} i_x, \tag{85}$$

where F is Faraday's constant (in C/mmol), representing the electric charge per mmol of ions with elementary charge. Note that the reason for the factor two in the denominator is that the valence of a Ca^{2+} ion is two, and the factor 10^{-15} is included in the numerator to convert the flux from unit amol/ms to unit mmol/ms. Moreover, the reason for the negative sign in (85) is that the positive direction of the single channel current by convention is from the inside to the outside of the cell, whereas the positive direction of the Ca^{2+} flux is defined to be from the outside to the inside of the cell. Note also that since the Na^+-Ca^{2+} exchanger exchanges three Na^+ ions for one Ca^{2+} ion, the flux of one Ca^{2+} ion through the exchanger represents the exchange of one charge instead of two, and a positive current out of the cell is associated with a flux of Ca^{2+} into the cell. Therefore (85) is replaced by

$$j_{\text{NaCa}} = \frac{10^{-15}}{F} i_{\text{NaCa}} \tag{86}$$

in this case.

Combining (83)–(86), we see that the total membrane fluxes may be written as

$$J_{\text{CaL}} = -\frac{\chi C_m}{2F} I_{\text{CaL}}, \qquad J_{\text{pCa}} = -\frac{\chi C_m}{2F} I_{\text{pCa}}, \tag{87}$$

$$J_{\text{bCa}} = -\frac{\chi C_m}{2F} I_{\text{bCa}}, \qquad J_{\text{NaCa}} = \frac{\chi C_m}{F} I_{\text{NaCa}}, \tag{88}$$

where

$$\chi = \frac{A}{10^{15} V_{\text{cell}}} \tag{89}$$

is the surface-to-volume ratio of the cell (in μm^{-1}).

Scaling the surface-to-volume ratio As explained above, we assume that the density of the membrane channels responsible for the Ca^{2+} fluxes may be different for different levels of maturity. This change in channel density is represented in the model by scaling the currents (see Section 2.2.1 of the paper), which will also affect the corresponding Ca^{2+} fluxes (87)–(88).

In addition, we assume that the geometry of the cells (i.e., the membrane area, A, and the cell volume, V_{cell}) may be different for different levels of maturity. From (87)–(90), we see that this change in geometry may be represented by scaling the surface-to-volume ratio, χ, by

$$\chi^{S_1} = (1 + \lambda_\chi)\chi^{S_2}, \tag{90}$$

where χ^{S_1} is the surface-to-volume ratio for maturity stage S_1, χ^{S_2} is the value for maturity stage S_2, and λ_χ is an adjustment factor for the surface-to-volume ratio.

S2.2 Notes on modeling Ca^{2+} dynamics

The inversion algorithm requires thousands of simulations testing different parameters representing geometrical properties and channel densities, either in terms of membrane channels or in terms of channels or buffers involved in the intracellular Ca^{2+} machinery. In order for the inversion to work properly, it is essential that the AP model is stable with respect to variations in the parameters. In particular, it is important that the simulation does not fail because of instabilities in the model.

Modeling the intracellular Ca^{2+} dynamics of cardiac cells has been a long-standing challenge and a very active field of research for at least 40 years; for reviews see e.g., [6, 7, 8, 9, 10]. Ca^{2+} dynamics are a complex time-dependent, 3D and highly non-linear problem. Mathematical models have attempted to represent the dynamics using a system of ordinary differential equations. Essentially, the goal of these models has been to remove the spatial variance and compute solutions that are spatially averaged and therefore merely depend on time. The main motivation for this strategy is to achieve models that are practical to work with in terms of computational complexity. However, the strategy has run into serious modeling challenges that have subsequently been addressed with ingenuity in numerous models (see e.g., [11, 12, 13, 14, 15, 16, 4, 17, 6]). Also spatial models (see e.g., [18, 19, 20, 21]) and homogenized spatial models (see e.g., [22, 23, 24, 25, 26]) have been applied, and while these models clearly capture the intricate dynamics more convincingly, this comes at a computational cost that renders them impractical for the purpose of this study and many other applications,

as tens of thousands of simulations with spatially resolved 3D models of the Ca^{2+} dynamics of cardiac cells is not computationally tractable at present. Below, we will discuss some important concepts involved in the intracellular Ca^{2+} dynamics of cardiac cells and some previously introduced modeling approaches for these dynamics.

S2.2.1 Ca^{2+}-induced Ca^{2+} release (CICR)

In the early phase of the upstroke of the AP, the membrane potential increases sufficiently for the voltage-sensitive dihydropyridine receptors (DHPR) to open the L-type Ca^{2+} channels on the membrane. Because of the huge gradient in the Ca^{2+} concentration between the intracellular and extracellular spaces, Ca^{2+} ions cross the membrane and flow into the cell. Inside the cell, the Ca^{2+} enters a tiny dyad (see Figure S1) located between the cell membrane and the sarcoplasmic reticulum (SR). Since the dyad is very small, the Ca^{2+} concentration increases rapidly and the increased concentration is sensed by the ryanodine receptors (RyRs) which in turn open and allow large amounts of Ca^{2+} to flow out of the SR. The increased Ca^{2+} concentration spreads by diffusion and recruits other RyRs to open and thus even more Ca^{2+} is poured into the bulk cytosolic space. This process is usually referred to as Ca^{2+}-induced Ca^{2+} release (CICR), and it takes place in many thousand local Ca^{2+} release units (CRUs) close to the cell membrane (see e.g., [27, 28, 29, 18]).

S2.2.2 High gain and graded release

The CICR is designed to provide both *high gain* and *graded release* (see e.g., [29, 6]). High gain means that, even when only a small amount of Ca^{2+} enters the cell through the cell membrane, this small amount leads to release of a much larger amount of Ca^{2+} from the SR. However, the release is also graded (see e.g., [30, 31, 27, 29, 6]) in the sense that the release of Ca^{2+} from the SR into the bulk cytosolic space depends continuously on the amount of Ca^{2+} flowing into the cell through the channels on the cell membrane. In other words, the amount of Ca^{2+} flowing into the cytosol during an AP is believed to be controlled by the flow through the membrane Ca^{2+} channels, even if most of the Ca^{2+} is released from the internal storage structures (the SR).

S2.2.3 Restoring the Ca^{2+} concentration

The AP is periodic and at the end of one cycle, all variables are brought back to the repolarized state of the cell. Ca^{2+} is pumped back to SR by the SERCA

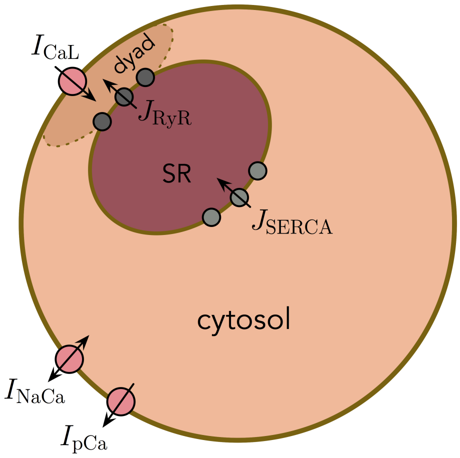

Figure S1: A prototypical sketch of a common pool model with Ca^{2+} flowing into the dyad through L-type Ca^{2+} channels; the RyR-channels are activated when the Ca^{2+} concentration increases, and Ca^{2+} is transported back to the SR from the cytosol via the SERCA pump and back to the extracellular space through the Na^+-Ca^{2+} exchanger and the Ca^{2+} pump on the cell membrane.

(sarcoplasmic reticulum Ca^{2+} ATPase) pump, and back to the extracellular space through the membrane Ca^{2+} pump and the Na^+-Ca^{2+} exchanger.

S2.2.4 Common pool models

A standard approach to modeling CICR is illustrated in Figure S1. Here, the dynamics of the many CRUs are modeled by one representative unit, hence all CRUs are assumed to be in the same state. In the model, Ca^{2+} enters the dyad through L-type Ca^{2+} channels, which leads to an increased dyadic Ca^{2+} concentration, and thus the RyRs open and Ca^{2+} leaves the SR. Models of the form illustrated in Figure S1 is referred to as *common pool models* and are characterized by the fact that Ca^{2+} released through the RyRs (from the SR) enters the same, small, dyadic space that Ca^{2+} enters through the L-type Ca^{2+} channel. It has been known for a long time (see [11]) that it is impossible to obtain graded release using stable common pool models. The problem is that when the release of Ca^{2+} from the SR has started, the release from the SR will itself cause an increased dyadic Ca^{2+} concentration, and release will continue until some inactivation mechanism of the release (e.g., a sufficiently decreased SR Ca^{2+} concentration [32]) kicks in. Consequently,

the release becomes an *all or nothing* process, depending only on whether the amount of Ca^{2+} entering the dyad through L-type Ca^{2+} channels is enough to trigger release. Therefore, graded release cannot be obtained using a model of the form given in Figure S1.

S2.2.5 Local control models

The difficulties associated with the common pool models can be circumvented by allowing many CRUs in the model (see e.g., [18, 19, 29, 20]). By introducing a large number of CRUs that are weakly coupled and where the release mechanisms are governed by stochastic Markov models, it is possible to achieve both high gain and graded release. Suppose there are $\sim$ 20.000 CRUs (as suggested in [20]) and every CRU has the elements illustrated in Figure S1 where the release mechanism of the L-type Ca^{2+} channel and the RyR are governed by Markov models. For simplicity we assume that the open probability of the L-type Ca^{2+} channels and the RyRs increases with increasing membrane potential and increasing dyadic Ca^{2+} concentration, respectively. Then, when the membrane potential increases slightly, the open probability of the L-type Ca^{2+} channels increases sufficiently for a few membrane channels to open, and thus Ca^{2+} will flow into the dyad of the associated CRUs. Locally, in these CRUs, the increased dyadic Ca^{2+} concentration will lead to increased open probability of the RyRs and when these channels open, the local SR of that particular CRU will be emptied. When the membrane potential increases more, the number of active CRUs will increase, and thus, the release will be graded by the membrane potential. So even if every single CRU is an all or nothing process, the integrated process is controlled by the membrane potential. Unfortunately, since these models requires a large number of CRUs, the computational cost of these models is prohibitive for our purposes.

S2.2.6 CICR in the base model

In the base model introduced in the current paper, we introduce two main modeling assumptions to obtain a model that exhibits both high gain and graded release without the high computational cost of local control models. First, the Ca^{2+} released from the SR is not released into the dyad, but is instead directed into a separate subsarcolemmal (SL) space. By directing the Ca^{2+} into this space, the Ca^{2+} entering the dyad though the membrane Ca^{2+} channels are clearly distinguishable from that released from the SR, and we avoid the graded-release problem associated with the common pool models. In addition, instead of inactivating the release from the SR by

a decreased SR Ca^{2+} concentration, we introduce an assumption that each channel can only release a certain amount of Ca^{2+} during an AP cycle, introduced because the SR Ca^{2+} concentration can potentially vary significantly for the large parameter changes considered in the inversion procedure. In Figure 4 of the paper, we observe that the model constructed from these assumptions exhibits both high gain and graded release for the immature and mature versions of the parameters.

S3 Details of the inversion procedure

S3.1 Definition of the cost function

In the inversion procedure, we consider a cost function of the form

$$H(\lambda, \varepsilon) = \sum_d \sum_j w_{d,j}(H_j(\lambda, \varepsilon, D_d))^2, \tag{91}$$

where H_j represent various differences between the data and the model solution (see Section 2.4.5 of the paper). Below, each of the cost function terms H_j are defined.

Note that the considered data from the hiPSC-CM microphysiological systems are traces measuring the membrane potential and the cytosolic Ca^{2+} concentration. As these measurements are optically obtained using voltage- and Ca^{2+}-sensitive dyes, some characteristics of the AP and Ca^{2+} transient cannot be sampled directly from the data, for instance the maximum and minimum values of the membrane potential and Ca^{2+} concentration. However, other biomarkers, like the AP duration, are readily obtained. When necessary for comparing simulation results (with units) and experimental data (unitless), experimental data values are mapped so that the maximum and minimum values of the membrane potential and the Ca^{2+} transient match the maximum and minimum values of the model solution.

S3.1.1 Action potential and Ca^{2+} transient durations

The terms in the cost function include terms for the differences in the AP and Ca^{2+} transient durations of the form

$$H_{\text{APD}p}(\lambda, \varepsilon, D_d) = \frac{|\text{APD}p(\lambda, \varepsilon, D_d) - \text{APD}p^*(D_d)|}{|\text{APD}p^*(D_d)|}, \tag{92}$$

$$H_{\text{CaD}p}(\lambda, \varepsilon, D_d) = \frac{|\text{CaD}p(\lambda, \varepsilon, D_d) - \text{CaD}p^*(D_d)|}{|\text{CaD}p^*(D_d)|}, \tag{93}$$

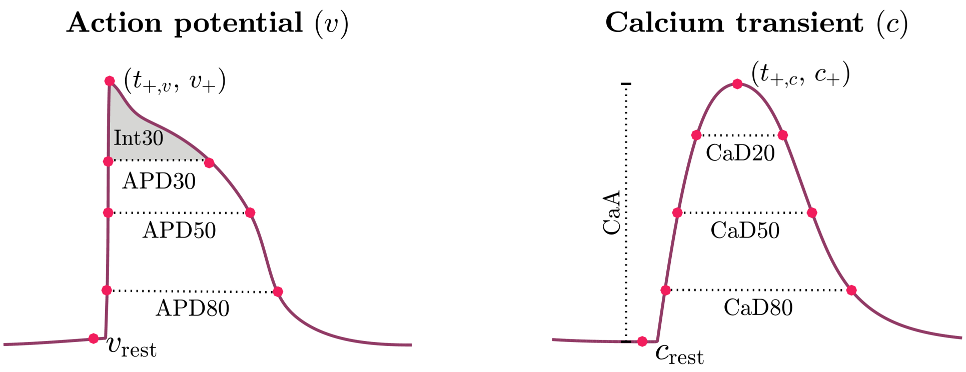

Figure S2: Illustration of some of the quantities used to define the terms of the cost function from the AP and Ca^{2+} transient.

for $p = 20, 25, ...75, 80$. Here, as an example, APD30 is defined as the time from the membrane potential is 30% below its maximum value during the upstroke of the AP, t_1, to the time at which the membrane potential again reaches a value 30% below its maximum value during the repolarization phase, t_2. APD30$(\lambda, \varepsilon, D_d)$ is the value obtained from the solution of the model given by the parameter vectors λ and ε for the drug dose D_d, while APD30$^*(D_d)$ is the value obtained from data for the drug dose D_d. The same notation, with a '$*$' marking the measured data values, is used for all the terms in the cost function. The Ca^{2+} transient durations, CaDp, are defined in the same manner as the AP durations.

S3.1.2 Integral of the membrane potential

Because the APDp values for low values of p may be difficult to obtain from the optical measurements due to noise, we also include a term that considers the integral of the membrane potential from t_1 to t_2 (see Figure S2). This term is defined as

$$H_{\text{Int30}}(\lambda, \varepsilon, D_d) = \frac{|\text{Int30}(\lambda, \varepsilon, D_d) - \text{Int30}^*(D_d)|}{|\text{Int30}^*(D_d)|}, \tag{94}$$

where Int30 is defined as

$$\text{Int30} = \int_{t_1}^{t_2} [v - v(t_1)]\, dt, \tag{95}$$

and v is the membrane potential. Note that the values of t_1 and t_2 are here the ones defined in the computation of APD30.

S3.1.3 Norm of the Ca^{2+} transient difference

As there is typically less noise in the data obtained from optical measurements of the Ca^{2+} transient as compared to optical measurements of the membrane potential, we also include a term for the discrete l^2-norm of the difference between the Ca^{2+} transient of the data and the model,

$$H_{\mathrm{Ca}}(\lambda, \varepsilon, D_d) = \frac{\|c(\lambda, \varepsilon, D_d) - c^*(D_d)\|_2}{\|c^*(D_d)\|_2}, \tag{96}$$

where c is the cytosolic Ca^{2+} concentration. When the data c^* is obtained from optical measurements, the timing of the Ca^{2+} transient relative to the stimulation time is not known. Therefore, the value of H_{Ca} is taken as the smallest value obtained when the timing of $c^*(D_d)$ may be adjusted to fit the timing of $c(\lambda, \varepsilon, D_d)$.

S3.1.4 Upstroke velocity

In order to capture information about the upstroke of the AP and Ca^{2+} transient, we also consider the terms

$$H_{\mathrm{dvdt}}(\lambda, \varepsilon, D_d) = \frac{\left| \left(\frac{dv(\lambda,\varepsilon,D_d)}{dt}\right)_{-20\mathrm{mV}} - \left(\frac{dv^*(D_d)}{dt}\right)_{-20\mathrm{mV}} \right|}{\left| \left(\frac{dv^*(D_d)}{dt}\right)_{-20\mathrm{mV}} \right|}, \tag{97}$$

$$H_{\mathrm{dcdt}}(\lambda, \varepsilon, D_d) = \frac{\left| \left(\frac{dc(\lambda,\varepsilon,D_d)}{dt}\right)_{\mathrm{max}} - \left(\frac{dc^*(D_d)}{dt}\right)_{\mathrm{max}} \right|}{\left| \left(\frac{dc^*(D_d)}{dt}\right)_{\mathrm{max}} \right|}, \tag{98}$$

where $\left(\frac{dv}{dt}\right)_{-20\mathrm{mV}}$ is the upstroke velocity of the AP obtained at $v = -20$ mV, and $\left(\frac{dc}{dt}\right)_{\mathrm{max}}$ is the maximal upstroke velocity of the Ca^{2+} transient. We use the upstroke velocity obtained at $v = -20$ mV instead of the maximal upstroke velocity to ensure that the value obtained in the model is not determined by the stimulus current. Note, however, that because of the noise in the optical measurements of the membrane potential, the H_{dvdt}-term is currently only included in the inversions used to determine a mature base model. In that case, the "experimental" data used for parameterization are generated by simulations and therefore dv/dt can accurately be computed.

S3.1.5 Ca^{2+} transient amplitude

Because the Ca^{2+} transient amplitude is one of the main characteristics permitting distinction between block of I_{CaL} as opposed to I_{NaL}, we also include

the term

$$H_{\text{CaA}}(\lambda, \varepsilon, D_d) = \frac{|\text{CaA}(\lambda, \varepsilon, D_d) - \text{CaA}^*(D_d)|}{|\text{CaA}^*(D_d)|}, \tag{99}$$

where CaA denotes the Ca^{2+} transient amplitude (see Figure S2).

Note, however, that the actual values of the Ca^{2+} transient amplitude are not known from the optical measurements, and only the relative differences of the amplitude between the control case and the different drug doses are known. Therefore, we do not include the H_{CaA}-term for the control case. For the non-zero drug doses, we define the data values $\text{CaA}^*(D_d)$ so that the relative difference between $\text{CaA}^*(D_d)$ and the amplitude in the control model is the same as the relative difference between the amplitude in the data for drug dose D_d and the control data. In other words, $\text{CaA}^*(D_d)$ is defined as

$$\text{CaA}^*(D_d) = \frac{\tilde{\text{CaA}}(D_d)}{\tilde{\text{CaA}}(D_0)} \text{CaA}(\lambda, \varepsilon, D_0), \tag{100}$$

where $\tilde{\text{CaA}}(D_d)$ and $\tilde{\text{CaA}}(D_0)$ are the unitless measured Ca^{2+} transient amplitudes for the drug dose D_d and the control case ($D_0 = 0$), respectively. Furthermore, $\text{CaA}(\lambda, \varepsilon, D_0)$ is the amplitude of the Ca^{2+} transient in the current control model given by the adjustment parameters λ.

S3.1.6 Maximum and resting values of the membrane potential and Ca^{2+} concentration

Where it is desirable to include information about the resting and maximum values of the membrane potential and/or the cytosolic Ca^{2+} concentration, we include terms of the form

$$H_{v,\text{rest}}(\lambda) = \frac{|v_{\text{rest}}(\lambda) - v^*_{\text{rest}}|}{|v^*_{\text{rest}}|}, \qquad H_{c,\text{rest}}(\lambda) = \frac{|c_{\text{rest}}(\lambda) - c^*_{\text{rest}}|}{|c^*_{\text{rest}}|}, \tag{101}$$

$$H_{v_+}(\lambda) = \frac{|v_+(\lambda) - v^*_+|}{|v^*_+|}, \qquad H_{c_+}(\lambda) = \frac{|c_+(\lambda) - c^*_+|}{|c^*_+|}, \tag{102}$$

$$H_{t_{+,v}}(\lambda) = \frac{|t_{+,v}(\lambda) - t^*_{+,v}|}{|t^*_{+,v}|}, \qquad H_{t_{+,c}}(\lambda) = \frac{|t_{+,c}(\lambda) - t^*_{+,c}|}{|t^*_{+,c}|}, \tag{103}$$

where v_{rest} and c_{rest} are the resting membrane potential and Ca^{2+} concentration, respectively, defined as the values obtained 10 ms before stimulation in the applied stimulation protocol. Similarly, v_+ and c_+ are the maximum values of the membrane potential and Ca^{2+} concentration, respectively, and $t_{+,v}$ and $t_{+,c}$ are the points in time at which these values are reached. Note that these terms are only included when the base model is fitted to the Grandi et al. model to define a mature base model.

S3.1.7 Information about individual currents

When the inversion procedure is used to define a default base model for mature and immature cells, information about the individual currents and fluxes is also included. These data are obtained from mathematical models of adult cardiomyocytes [1] and hiPSC-CMs [3], and are represented by cost function terms of the form

$$H_{I_x}(\lambda) = \frac{\|I_x(\lambda) - I_x^*\|_2}{\|I_x^*\|_2}, \qquad H_{I_x^{\max}}(\lambda) = \frac{|I_x^{\max}(\lambda) - I_x^{*,\max}|}{|I_x^{*,\max}|}, \tag{104}$$

for each of the considered currents or fluxes, x. Here, $\|\cdot\|_2$ is the discrete l^2-norm, and $I_x^{\max}$ is defined as $I_x^{\max} = \max(|I_x|)$.

S3.1.8 Ca^{2+} balance

We wish to select values of λ so that the resulting control model does not exhibit large degrees of drift in the intracellular Ca^{2+} concentrations. Therefore, we include a Ca^{2+} balance term of the form

$$H_{\mathrm{Ca},b}(\lambda) = \frac{1}{b}\left|\int_0^T (J_{\mathrm{CaL}}(\lambda) + J_e^{sl}(\lambda))dt\right|, \tag{105}$$

which is zero if the amount of Ca^{2+} entering the cell equals the amount of Ca^{2+} leaving the cell. The main term is here the absolute value of the integral of the sum of the J_{CaL} and J_e^{sl} fluxes of the model over the simulated time interval, and b is a scaling factor set equal to 0.1 mM in our simulations.

S3.1.9 Regularization of adjustment factors

In cases where several choices of parameters λ and ε fit the data equally well, we wish to choose values of λ and ε close to zero. We therefore include the regularization terms

$$H_\varepsilon(\varepsilon) = \sum_{i\in S_\varepsilon}\left(\frac{\varepsilon_i}{\bar{\varepsilon}}\right)^2, \qquad H_\lambda(\lambda) = \sum_{i\in S_{\lambda^\star}} \lambda^2. \tag{106}$$

Here, $\bar{\varepsilon} = \frac{1}{\bar{D}}$, where $\bar{D}$ is the median of the non-zero drug doses included in the data set. Furthermore, S_ε is the set of indices for all the individual ε-factors, and $S_{\lambda^\star}$ is the set of indices for the λ-values we want to remain as close as feasible to the default base model during the inversion. In the inversions reported in the paper, this set consists of the indices for λ_{CaL} and λ_{Kr}, as the size of these currents is based on measurements of hiPSC-CMs (see Section 3.1.2 of the paper) and because we are especially interested in obtaining reasonable, physiological values for these currents, as we are investigating the drug effects on these currents.

S3.2 Specification of the cost function weights

The choice of terms included in the cost function depends on the specific application of the inversion procedure. In particular, for inversions of data from optical measurements (and for inversions of simulated drugs), we include the terms H_{APD30}, H_{APD50}, H_{APD80}, $H_{\text{CaD20}} - H_{\text{CaD80}}$, H_{int30}, H_{dcdt}, H_{Ca}, H_{ε} and H_{λ}. We only include three APD values, but 13 CaD values as quality of the Ca^{2+} data is generally better than that of the membrane potential data. To make up for the large number of CaD-terms as compared to APD-terms, the weight of the CaD-terms are set to 0.5, while the APD-terms are given the weight 1 (the exception being that the weight of the APD80 and CaD80 terms are each set to 5). The upstroke velocity of the AP is not included because of the high noise level in the membrane potential data.

Furthermore, for the control case, the term $H_{\text{Ca},b}$ with weight 1 is included, and for the drug doses, H_{CaA} is included with weight 10. The large weight in this case is due to the fact that this is one of the most important characteristics for distinguishing between block of I_{CaL} and I_{NaL}. We also include the regularization terms H_{ε} and H_{λ} with weights, 0.01 and 10, respectively. All the ε-parameters are included in the ε-regularization, but only λ_{CaL} and λ_{Kr} are included in the λ-regularization as explained above.

In addition, the weight of all the cost function terms (except $H_{\text{Ca},b}$) are for the control case multiplied by the number of non-zero doses included in the data set. This is done because a good fit for the control model is essential for being able to use the model to estimate drug effects.

In the inversions aiming to define default values for the adult and hiPSC-CM base models, additional terms, e.g., terms for the individual currents, are also included in the cost function. This is specified in more detail in Sections 3.1.1 and 3.1.2 of the paper.

S4 Supplementary tables

Parameter	Final value	Initial value	Parameter	Final value	Initial value
g_{Na} (mS/μF)	12.6	11.0	$\bar{I}_{\text{NaCa}}$ (μA/μF)	4.9	4.8
g_{NaL} (mS/μF)	0.025	0.02	$\bar{I}_{\text{pCa}}$ (μA/μF)	0.068	0.07
g_{to} (mS/μF)	0.27	0.12	$\bar{J}_{\text{SERCA}}$ (mM/ms)	0.00024	0.00025
g_{Kr} (mS/μF)	0.025	0.03	α_{RyR} (ms^{-1})	0.0075	0.01
g_{Ks} (mS/μF)	0.003	0.002	α_d^c (ms^{-1})	0.0017	0.0025
g_{K1} (mS/μF)	0.37	0.43	α_{sl}^c (ms^{-1})	0.15	0.15
g_{f} (mS/μF)	0.0001	0.0001	α_n^s (ms^{-1})	0.012	0.01
g_{bCl} (mS/μF)	0.007	0.008	B_{tot}^c (mM)	0.07	0.08
$\bar{I}_{\text{NaK}}$ (μA/μF)	1.8	1.5	B_{tot}^d (mM)	1.2	1.3
g_{CaL} (nL/(μF ms))	0.12	0.1	B_{tot}^{sl} (mM)	0.9	1.0
g_{bCa} (mS/μF)	0.00055	0.0005	B_{tot}^s (mM)	27	29

Table S9: Hand-tuned initial guesses and the values returned by the inversion procedure defining the adult base model. The cost function includes all the terms specified in Section S3.1, except for the regularization terms. For the cost function terms involving information about currents and fluxes, we have included I_{Na}, I_{CaL}, I_{to}, I_{Kr}, I_{Ks}, I_{K1}, I_{NaCa}, I_{pCa}, and I_{bCa}, as well as the fluxes J_{RyR} and J_{SERCA} from the Grandi et al. model [1].

S5 Supplementary figures

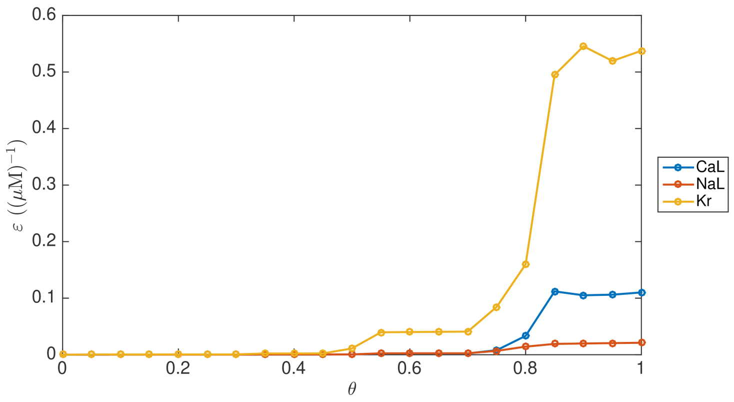

Figure S3: Example of how the optimal ε-values gradually move from zero to the optimal values for the data as θ is increased from zero to one in the continuation-based inversion procedure.

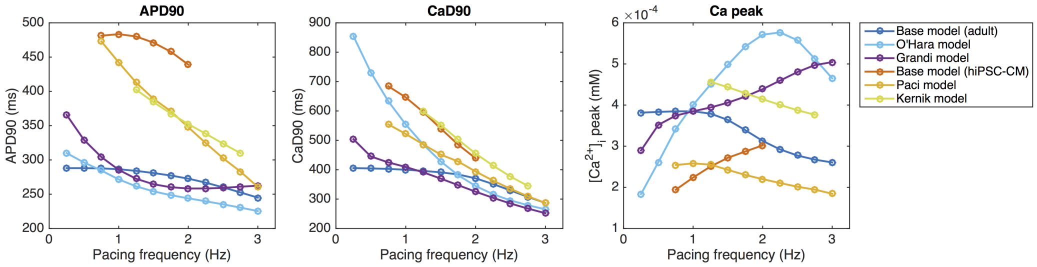

Figure S4: Frequency dependence of APD90, CaD90 and peak cytosolic Ca^{2+} concentration in the adult and hiPSC-CM versions of the base model, as well as in the Grandi [1] and O'Hara [2] models for adult cardiomyocytes and the Paci [3] and Kernik [33] models for hiPSC-CMs. For definitions of APD90 and CaD90, see Section S3.1.1. For all the models, the simulations are run for 200 AP cycles before recording the AP and Ca^{2+} transients whose properties are displayed in the plots. Note that because APD90 $\approx$ 500 ms for the default hiPSC-CM base model, pacing frequencies above 2 Hz (corresponding to cycle lengths below 500 ms) imply stimulation before the final repolarization of the action potential. Therefore, we do not include data for pacing frequencies above 2 Hz for the default hiPSC-CM base model.

References

[1] Eleonora Grandi, Francesco S Pasqualini, and Donald M Bers. A novel computational model of the human ventricular action potential and Ca transient. *Journal of Molecular and Cellular Cardiology*, 48(1):112–121, 2010.

[2] Thomas O'Hara, László Virág, András Varró, and Yoram Rudy. Simulation of the undiseased human cardiac ventricular action potential: Model formulation and experimental validation. *PLoS Computational Biology*, 7(5):e1002061, 2011.

[3] Michelangelo Paci, Jari Hyttinen, Katriina Aalto-Setälä, and Stefano Severi. Computational models of ventricular-and atrial-like human induced pluripotent stem cell derived cardiomyocytes. *Annals of Biomedical Engineering*, 41(11):2334–2348, 2013.

[4] Thomas R Shannon, Fei Wang, José Puglisi, Christopher Weber, and Donald M Bers. A mathematical treatment of integrated Ca dynamics within the ventricular myocyte. *Biophysical Journal*, 87(5):3351–3371, 2004.

[5] James P Keener and James Sneyd. *Mathematical Physiology*. Springer, 2009.

[6] Geneviève Dupont, Martin Falcke, Vivien Kirk, and James Sneyd. *Models of calcium signalling*, volume 43. Springer, 2016.

[7] Eduardo Ríos. Calcium-induced release of calcium in muscle: 50 years of work and the emerging consensus. *The Journal of General Physiology*, 150(4):521–537, 2018.

[8] Eric A Sobie, George SB Williams, and WJ Lederer. Ambiguous interactions between diastolic and SR Ca^{2+} in the regulation of cardiac Ca^{2+} release. *The Journal of General Physiology*, pages jgp–201711814, 2017.

[9] David A Eisner, Jessica L Caldwell, Kornél Kistamás, and Andrew W Trafford. Calcium and excitation-contraction coupling in the heart. *Circulation Research*, 121(2):181–195, 2017.

[10] David A Eisner. Ups and downs of calcium in the heart. *The Journal of Physiology*, 596(1):19–30, 2018.

[11] Michael D Stern. Theory of excitation-contraction coupling in cardiac muscle. *Biophysical Journal*, 63(2):497–517, 1992.

[12] Anders Nygren, Céline Fiset, Ludwik Firek, John W Clark, Douglas S Lindblad, Robert B Clark, and Wayne R Giles. Mathematical model of an adult human atrial cell: the role of K^+ currents in repolarization. *Circulation Research*, 82(1):63–81, 1998.

[13] Semahat S Demir, John W Clark, C Richard Murphey, and Wayne R Giles. A mathematical model of a rabbit sinoatrial node cell. *American Journal of Physiology-Cell Physiology*, 266(3):C832–C852, 1994.

[14] Socrates Dokos, Branko Celler, and Nigel Lovell. Ion currents underlying sinoatrial node pacemaker activity: a new single cell mathematical model. *Journal of Theoretical Biology*, 181(3):245–272, 1996.

[15] José L Puglisi and Donald M Bers. LabHEART: an interactive computer model of rabbit ventricular myocyte ion channels and Ca transport. *American Journal of Physiology-Cell Physiology*, 281:2049–2060, 2001.

[16] Eric A Sobie, Keith W Dilly, Jader dos Santos Cruz, W Jonathan Lederer, and M Saleet Jafri. Termination of cardiac Ca^{2+} sparks: an investigative mathematical model of calcium-induced calcium release. *Biophysical Journal*, 83(1):59–78, 2002.

[17] Juan G Restrepo, James N. Weiss, and Alain Karma. Calsequestrin-mediated mechanism for cellular calcium transient alternans. *Biophysical Journal*, 95(8):3767–3789, October 2008.

[18] Michael D Stern, Gonzalo Pizarro, and Eduardo Ríos. Local control model of excitation–contraction coupling in skeletal muscle. *The Journal of General Physiology*, 110(4):415–440, 1997.

[19] Michael D Stern, Long-Sheng Song, Heping Cheng, James SK Sham, Huang Tian Yang, Kenneth R Boheler, and Eduardo Ríos. Local control models of cardiac excitation–contraction coupling: a possible role for allosteric interactions between ryanodine receptors. *The Journal of General Physiology*, 113(3):469–489, 1999.

[20] Michael Nivala, Enno de Lange, Robert Rovetti, and Zhilin Qu. Computational modeling and numerical methods for spatiotemporal calcium cycling in ventricular myocytes. *Frontiers in Physiology*, 3(114), 2012.

[21] Johan Hake, Andrew G Edwards, Zeyun Yu, Peter M Kekenes-Huskey, Anushka P Michailova, J Andrew McCammon, Michael J Holst,

Masahiko Hoshijima, and Andrew D McCulloch. Modelling cardiac calcium sparks in a three-dimensional reconstruction of a calcium release unit. *Journal of Physiology*, 590(18):4403–4422, 2012.

[22] Pranay Goel, James Sneyd, and Avner Friedman. Homogenization of the cell cytoplasm: the calcium bidomain equations. *Multiscale Modeling & Simulation*, 5(4):1045–1062, 2006.

[23] Yuhui Cheng, Zeyun Yu, Masahiko Hoshijima, Michael J Holst, Andrew D McCulloch, J Andrew McCammon, and Anushka P Michailova. Numerical analysis of Ca^{2+} signaling in rat ventricular myocytes with realistic transverse-axial tubular geometry and inhibited sarcoplasmic reticulum. *PLoS Computational Biology*, 6(10):e1000972, 2010.

[24] Pawel Swietach, Kenneth W Spitzer, and Richard D Vaughan-Jones. Modeling calcium waves in cardiac myocytes: importance of calcium diffusion. *Frontiers in Bioscience*, 15:661–680, 2010.

[25] Aslak Tveito, Glenn T Lines, Johan Hake, and Andrew G Edwards. Instabilities of the resting state in a mathematical model of calcium handling in cardiac myocytes. *Mathematical Biosciences*, 236(2):97–107, 2012.

[26] Zhilin Qu, Gang Hu, Alan Garfinkel, and James N Weiss. Nonlinear and stochastic dynamics in the heart. *Physics Reports*, 543(2):61–162, 2014.

[27] Alexandre Fabiato. Calcium-induced release of calcium from the cardiac sarcoplasmic reticulum. *American Journal of Physiology-Cell Physiology*, 245(1):C1–C14, 1983.

[28] Mark B Cannell, Heping Cheng, and William J Lederer. The control of calcium release in heart muscle. *Science*, 268(5213):1045–1049, 1995.

[29] John J Rice, M Saleet Jafri, and Raimond L Winslow. Modeling gain and gradedness of Ca^{2+} release in the functional unit of the cardiac diadic space. *Biophysical Journal*, 77(4):1871–1884, 1999.

[30] LL Costantin and SR Taylor. Graded activation in frog muscle fibers. *The Journal of General Physiology*, 61(4):424–443, 1973.

[31] Makoto Endo. Calcium release from the sarcoplasmic reticulum. *Physiological Reviews*, 57(1):71–108, 1977.

[32] Eric A Sobie, M Saleet Jafri, and WJ Lederer. Models of cardiac Ca^{2+}-induced Ca^{2+} release and Ca^{2+} sparks. In Martin Falcke and Dieter Malchow, editors, *Understanding Calcium Dynamics. Experiments and Theory.*, chapter 6, pages 97–118. Springer-Verlag, Berlin Heidelberg, 2010.

[33] Divya C Kernik, Stefano Morotti, Hao Di Wu, Priyanka Garg, Henry J Duff, Junko Kurokawa, Jose Jalife, Joseph C Wu, Eleonora Grandi, and Colleen E Clancy. A computational model of induced pluripotent stem-cell derived cardiomyocytes incorporating experimental variability from multiple data sources. *The Journal of Physiology*, 597(17):4533–4594, 2019.
